# Locomotor and postural diversity among reptiles viewed through the prism of femoral microanatomy: Palaeobiological implications for some Permian and Mesozoic taxa

**DOI:** 10.1111/joa.13833

**Published:** 2023-02-17

**Authors:** Jordan Gônet, Jérémie Bardin, Marc Girondot, John R. Hutchinson, Michel Laurin

**Affiliations:** ^1^ Centre de recherche en paléontologie – Paris, UMR 7207 Sorbonne Université, Muséum national d'histoire naturelle, Centre national de la recherche scientifique Paris France; ^2^ Laboratoire écologie, systématique et évolution, UMR 8079 AgroParisTech, Université Paris‐Saclay, Centre national de la recherche scientifique Orsay France; ^3^ Structure and Motion Laboratory, Royal Veterinary College Department of Comparative Biomedical Sciences Hatfield UK

**Keywords:** locomotion, posture, reptile, femur, microanatomy, functional morphology, palaeobiology

## Abstract

The water‐to‐land transition by the first tetrapod vertebrates represents a key stage in their evolution. Selection pressures exerted by this new environment on animals led to the emergence of new locomotor and postural strategies that favoured access to different ecological niches and contributed to their evolutionary success. Today, amniotes show great locomotor and postural diversity, particularly among Reptilia, whose extant representatives include parasagittally locomoting erect and crouched bipeds (birds), sub‐parasagittal ‘semi‐erect’ quadrupeds (crocodylians) and sprawling quadrupeds (squamates and turtles). But the different steps leading to such diversity remain enigmatic and the type of locomotion adopted by many extinct species raises questions. This is notably the case of certain Triassic taxa such as *Euparkeria* and *Marasuchus*. The exploration of the bone microanatomy in reptiles could help to overcome these uncertainties. Indeed, this locomotor and postural diversity is accompanied by great microanatomical disparity. On land, the bones of the appendicular skeleton support the weight of the body and are subject to multiple constraints that partly shape their external and internal morphology. Here we show how microanatomical parameters measured in cross‐section, such as bone compactness or the position of the medullocortical transition, can be related to locomotion. We hypothesised that this could be due to variations in cortical thickness. Using statistical methods that take phylogeny into account (phylogenetic flexible discriminant analyses), we develop different models of locomotion from a sample of femur cross‐sections from 51 reptile species. We use these models to infer locomotion and posture in 7 extinct reptile taxa for which they remain debated or not fully clear. Our models produced reliable inferences for taxa that preceded and followed the quadruped/biped and sprawling/erect transitions, notably within the Captorhinidae and Dinosauria. For taxa contemporary with these transitions, such as *Terrestrisuchus* and *Marasuchus*, the inferences are more questionable. We use linear models to investigate the effect of body mass and functional ecology on our inference models. We show that body mass seems to significantly impact our model predictions in most cases, unlike the functional ecology. Finally, we illustrate how taphonomic processes can impact certain microanatomical parameters, especially the eccentricity of the section, while addressing some other potential limitations of our methods. Our study provides insight into the evolution of enigmatic locomotion in various early reptiles. Our models and methods could be used by palaeontologists to infer the locomotion and posture in other extinct reptile taxa, especially when considered in combination with other lines of evidence.

## Graphical abstract

Reptiles show great diversity of locomotion and posture. We employed phylogenetically informed statistical methods to explore their bone microanatomy. We used our models to infer the locomotion of extinct reptiles that document the different episodes of postural diversification in this clade.

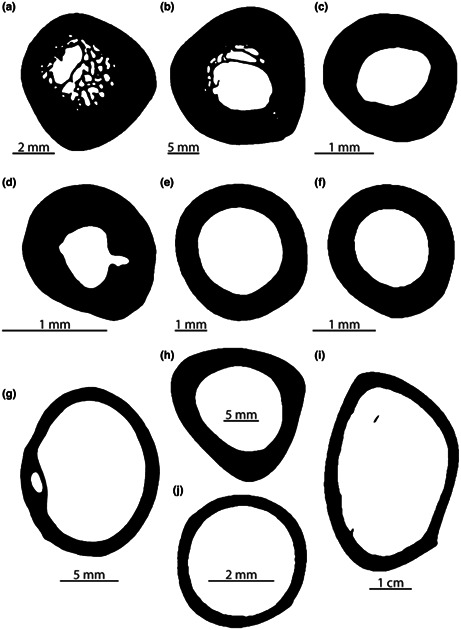

## 1 INTRODUCTION

The transition to land during the Devonian period was a key stage in the evolution of tetrapod vertebrates. A key element in this colonisation was the innovation of the limb (Hall, 2007). Composed of three articulated segments (the stylopod [humerus and femur], the zeugopod [radius/ulna and tibia/fibula] and the autopod [hand and foot]), limbs first evolved in aquatic organisms such as *Acanthostega* and were presumably used for locomotion in shallow waters and to rest on the bottom (Coates, 1996; Laurin, 2010; Molnar et al., 2021). From this primordial limb, under the action of new constraints inherent to the terrestrial environment, particularly related to gravity, the first inhabitants of land evolved novel postural strategies favouring access to different ecological niches and contributing to the evolutionary success of tetrapods.

On land, the bones of the appendicular skeleton support the weight of the body and are subject to forces that partly shape their external and internal morphology. Indeed, bone is a living tissue. It undergoes perpetual modelling and remodelling (shape change to maintain strength and to repair microdamage, respectively) under the action of osteoblasts and osteoclasts which participate respectively in the formation and destruction of this tissue (Currey, 2013). This process is subject not only to fine molecular control but also to mechanical regulation in order to maintain or increase bone strength (Robling et al., 2006). In the case of a load‐bearing bone, bone trabeculae (network of bony columns and plates constituting the cancellous bone) tend to orient themselves along the main lines of force: this is known as Wolff's law or the trajectorial theory (Wolff, 1893). Many examples of bone modelling and remodelling exist in the literature. For example, Cubo et al. (2015) reported the first case of biomechanically adaptive bone modelling in a non‐avian dinosaur. In *Maiasaura*, following a fracture of the fibula, the tibia was subjected to compensatory overstress resulting in periosteal bone modelling with the formation of a bony outgrowth in the direction of the presumed stress. In fact, the location of the outgrowth was age‐dependent: it was located anterolaterally in supposedly bipedal *Maiasaura* juveniles and posteriorly in likely quadrupedal adults. This allowed the inference of a transition from bipedalism to quadrupedalism during ontogeny. More recently, Mitchell et al. (2017) showed that the amount of Haversian bone (remodelled bone) in the furcula of birds differed between taxa employing high‐frequency flapping flight and those employing another type of flight. Because the furcula is subjected to greater depression forces in the former taxa, the study hypothesised that these greater forces may be one reason for greater remodelled tissue in these birds.

The first terrestrial vertebrates were quadrupedal with a sprawling limb posture, that is the stylopod was held horizontally with the distal end pointing laterally (Bakker, 1971; Charig, 1972). A recent study combining paleoichnology (the study of ancient tracks) and robotics supported this inference with quantitative methods for the first time (Nyakatura et al., 2019). Extant taxa present a great diversity of postures and locomotor modes associated with morphological and microanatomical disparity. This diversity is especially true for the clade Reptilia, which includes parasagittally locomoting erect and crouched bipeds (birds), sub‐parasagittally locomoting ‘semi‐erect’ quadrupeds (crocodylians) and more sprawling quadrupeds as extant representatives (Blob & Biewener, 2001; Gatesy & Biewener, 1991; Reilly & Elias, 1998).

The first parasagittally locomoting erect bipedal amniotes evolved convergently during the Triassic Period in the archosaurian lineages Avemetatarsalia and Pseudosuchia. Parasagittally locomoting erect bipedalism is often cited as a key element in the success of Avemetatarsalia (Kubo & Kubo, 2012; Pintore et al., 2022). However, the steps that led to this bipedal state remain enigmatic and more conclusively determining the mode of locomotion adopted by many taxa that lived at this time such as the archosauriform *Euparkeria*, the crocodylomorph *Terrestrisuchus* or the dinosauromorph *Marasuchus* involve considerable obstacles and ambiguities (e.g. Bishop et al., 2020). Ornithopod dinosaurs are another good example of the challenge palaeontologists sometimes face in elucidating the limb posture of animals for which only bony remains and fossil trackways exist. Ornithopods are a group of herbivorous dinosaurs that proliferated during the Mesozoic Era. They originated in the Early Jurassic about 200 million years ago and disappeared at the Cretaceous/Paleogene boundary (~66 Ma). However, the question of their quadrupedal/bipedal stance, and whether or not there was a transition from bipedalism to quadrupedalism during ontogeny, remains debated (Barrett & Maidment, 2017; Dilkes, 2001; Galton, 1970; Norman, 1980; Wosik et al., 2017).

While many studies have already highlighted the link between lifestyle (aquatic to terrestrial) and bone microanatomy (Amson et al., 2014; Canoville & Laurin, 2009, 2010; Cooper et al., 2016; Germain & Laurin, 2005; Houssaye & Botton‐Divet, 2018; Houssaye, Sander, et al., 2016; Ibrahim et al., 2014; Klein et al., 2016; Kriloff et al., 2008; Laurin et al., 2011; Nakajima et al., 2014; Quemeneur et al., 2013), fewer have focused on the link between limb posture and microanatomy (Bishop, Hocknull, Clemente, Farke, et al., 2018b, 2018c; Bishop, Hocknull, Clemente, Hutchinson, Barrett, et al., 2018; Houssaye, Waskow, et al., 2016; Plasse et al., 2019; Wagstaffe et al., 2022). In line with these latter works, this study aims to improve our understanding of the relationships between posture and bone microstructure in reptiles. Using mid‐diaphyseal cross‐sections of extant and extinct reptile femora, we test for geometric and microanatomical differences between taxa that reflect locomotion and posture. Typically, regardless of allometric and environmental factors (which will be considered in detail), we would expect bipeds to have greater bone compactness (proportion of bone tissue in a skeletal element) than quadrupeds, as body mass is more evenly distributed in the latter (two weight‐bearing limbs vs four). We use the data we obtain to construct phylogenetically informed models linking locomotion, posture and microanatomy, built on an extant and extinct sample, which then allow us to infer the posture of Palaeozoic and Mesozoic amniotes that document various phases of posture diversification in that clade.

## 2 MATERIALS AND METHODS

### 2.1 Biological sample

To train our statistical models, the femur of a large number of specimens and taxa from Reptilia has been included: 53 adult individuals from 51 species (37 archosaurs, 12 squamates and 2 turtles). Of these species, 6 taxa are extinct (but of ‘known’ posture) to give ‘phylogenetic depth’ to our sample. The latter include *Masiakasaurus knopfleri*, *Allosaurus fragilis*, *Tyrannosaurus rex*, *Dinornis* sp., *Pezophaps solitaria* and *Raphus cucullatus* (Table 1; Supporting Information). All of these are theropod dinosaurs with a parasagittally locomoting erect bipedal stance (Hutchinson & Gatesy, 2001). We constructed our sample to be as representative as possible of the phylogenetic spectrum of Reptilia and to optimise coverage of the range of locomotor and postural diversity of the clade. We used our models to infer the posture of 7 extinct taxa of interest: *Labidosaurus hamatus*, *Euparkeria capensis*, *Terrestrisuchus gracilis*, *Marasuchus lilloensis*, *Plateosaurus engelhardti*, *Dysalotosaurus lettowvorbecki* and an indeterminate hypsilophodontid (Table 2; Supporting Information).

**TABLE 1:** List of the femora used to build the inference models presented in this study.

Taxon		Collection number	Locomotor mode	Posture	Functional ecology	Mass (g)	Femoral cross‐section	CT resolution (μm)
Accipitridae	*Gypaetus barbatus*	MNHN‐ZO‐AC‐1993‐52	B	C	Te	5606.042	CT scan	30
Anatidae	*Anas superciliosa*	UMZC 222.a	B	C	Aq	981	CT scan	31
	*Anser albifrons*	UMZC 242.e	B	C	Aq	2387.5	CT scan	37
	*Branta bernicla*	UMZC 246.f	B	C	Aq	1347.25	CT scan	36
	*Cereopsis novaehollandiae*	UMZC 242.aa	B	C	Aq	3770	CT scan	51
	*Chenonetta jubata*	UMZC 246.g	B	C	Aq	812.5	CT scan	29
	*Cygnus olor*	RVC	B	E	Aq	10230	CT scan	60
	*Somateria mollissima*	UMZC 704	B	C	Aq	2092	CT scan	36
Alligatoridae	*Alligator mississippiensis*	MNHN‐ZA‐AC‐1945‐54	Q	SE	Aq	62000	CT scan	46
	*Caiman crocodilus*	MNHN‐ZA‐AC‐1910‐87	Q	SE	Aq	10900	CT scan	30
Allosauridae†	*Allosaurus fragilis* †	DNM 2560	B	E	Te	1820150	CT scan	549
Apterygidae	*Apteryx australis*	UMZC 378.s	B	C	Te	2600	CT scan	61
	*Apteryx haastii*	UMZC 378.p	B	C	Te	2409	CT scan	44
	*Apteryx owenii*	UMZC 378.iii	B	C	Te	1200	CT scan	46
Casuariidae	*Casuarius casuarius*	MNHN‐ZO‐AC‐1946‐72	B	E	Te	44000	CT scan	57
	*Dromaius novaehollandiae*	YPM 2128	B	E	Te	36200	CT scan	186
Columbidae	*Columba livia*	RVC	B	C	Te	320	CT scan	25
	*Pezophaps solitaria* †	YPM 1154	B	E	Te	14000	CT scan	19
	*Raphus cucullatus* †	YPM 2064	B	E	Te	14000	CT scan	19
Crocodylidae	*Crocodylus niloticus*	MNHN‐ZA‐AC‐1963‐22	Q	SE	Aq	94200	CT scan	57
Cuculidae	*Geococcyx californianus*	UMZC 429.p	B	C	Te	376	CT scan	32
Dinornithidae†	*Dinornis* sp.†	YPM 421	B	E	Te	173500	CT scan	285
Megapodiidae	*Alectura lathami*	YPM 379	B	C	Te	2330	CT scan	188
Noasauridae†	*Masiakasaurus knopfleri* †	FMNH PR 2117	B	E	Te	18849.1	CT scan	188
Numididae	*Numida meleagris*	PJB	B	C	Te	1375	CT scan	55
Phasianidae	*Afropavo congensis*	YPM 6658	B	C	Te	1149.25	CT scan	188
	*Argusianus argus*	YPM 2100	B	C	Te	2280.5	CT scan	188
	*Synoicus ypsilophorus*	UMZC 405.a	B	C	Te	107.5	CT scan	5
	*Dendragapus obscurus*	YPM 11600	B	C	Te	1059	CT scan	188
	*Gallus* sp.	PJB	B	C	Te	828.9	CT scan	37
	*Meleagris gallopavo*	RVC	B	C	Te	5811	CT scan	72
	*Phasianus colchicus*	YPM 7778	B	C	Te	1043.75	CT scan	188
Rheidae	*Rhea americana*	MNHN‐ZO‐AC‐1876‐730	B	E	Te	23000	CT scan	57
Sagittariidae	*Sagittarius serpentarius*	YPM 1797	B	C	Te	3900	CT scan	188
Struthionidae	*Struthio camelus*	RVC	B	E	Te	109250	CT scan	390
Tinamidae	*Eudromia elegans*	MNHN‐ZO‐AC‐1905‐31	B	C	Te	678	CT scan	24
	*Eudromia elegans*	UMZC 404.e	B	C	Te	678	CT scan	188
Tyrannosauridae†	*Tyrannosaurus rex* †	MOR 1125	B	E	Te	7000000	CT scan	1178
Sphenodontidae	*Sphenodon punctatus*	uf:herp:14110^a^	Q	S	Fo	430	CT scan	74
	*Sphenodon punctatus*	ummz:herps:40651^a^	Q	S	Fo	430	CT scan	105
Agamidae	*Chlamydosaurus kingii*	ypm:vz:ypm herr 010336^a^	FB	S	Ar	449.125	CT scan	127
Corytophanidae	*Basiliscus basiliscus*	MNHN‐ZA‐AC‐1888‐124	FB	S	Ar	225	CT scan	15
	*Basiliscus vittatus*	MNHN‐ZA‐AC‐1883‐1830	FB	S	Ar	60.87	CT scan	15
Eublepharidae	*Coleonyx elegans*	ummz:herps:125878^a^	Q	S	Te	11.2	CT scan	56
Iguanidae	*Cyclura cornuta*	MNHN‐ZA‐AC‐1907‐107	Q	S	Te	16578.115	CT scan	34
	*Iguana iguana*	MNHN‐ZA‐AC‐1974‐129	Q	S	Ar	1530	CT scan	34
Phrynosomatidae	*Phrynosoma cornutum*	MNHN‐ZA‐AC‐1893‐662	Q	S	Te	27.335	CT scan	11
	*Urosaurus bicarinatus*	Unnumbered specimen	Q	S	Ar	3.415	Histological section	
Scincidae	*Tiliqua scincoides*	MNHN‐ZA‐AC‐1898‐285	Q	S	Te	496.4	CT scan	15
Varanidae	*Varanus gouldii*	MNHN‐ZA‐AC‐1889‐62	Q	S	Fo	671.92	CT scan	15
	*Varanus griseus*	MNHN‐ZA‐AC‐1920‐146	Q	S	Fo	821.1	Histological section	
Chelydridae	*Chelydra serpentina*	MNHN‐ZA‐AC‐1897‐255	Q	S	Aq	5170	CT scan	24
Testudinidae	*Chelonoidis carbonaria*	MNHN‐ZA‐AC‐1877‐404	Q	S	Te	2000	CT scan	30

Abbreviations: Aq, semi‐aquatic; Ar, arboreal; B, biped; C, parasagittally locomoting crouched; E, erect; FB, facultative biped; Fo, fossorial; Q, quadruped; S, sprawling; SE, 'semi‐erect'; Te, terrestrial.

^a^ Data collected on https://www.morphosource.org.

^†^ Indicates extinct taxa.

**TABLE 2:** List of the extinct taxa for which we inferred the posture.

Taxon		Collection number	Femoral cross‐section	CT resolution (μm)
Dryosauridae†	*Dysalotosaurus lettowvorbecki* †	GPIT/RE/3588	Histological section	
Hypsilophodontidae†	Indet. hypsilophodontid†	NMV P221151	Histological section	
Plateosauridae†	*Plateosaurus engelhardti* †	SMNS F 29	Histological section	
Dinosauriformes†	*Marasuchus lilloensis* †	PVL 3870	CT scan	23
Crocodylomorpha†	*Terrestrisuchus gracilis* †	NHMUK 72‐1	CT scan	16
Euparkeriidae†	*Euparkeria capensis* †	SAM PK 5867	CT scan	27
Captorhinidae†	*Labidosaurus hamatus* †	CM 73371	Histological section	

^†^ Indicates extinct taxa.

### 2.2 Locomotor modes

While the terms biped and quadruped refer to an animal moving respectively on two and four limbs, much like the sprawling‐to‐erect continuum of limb postures they do not correspond to well‐identified functional categories. Indeed, some bipeds can occasionally adopt a quadrupedal mode of locomotion (MOL) and vice versa. This is referred to as facultative quadrupedalism and bipedalism, respectively (Grinham et al., 2019; Hutchinson & Gatesy, 2001).

In the context of this study, any animal for which bipedalism is the exclusive MOL on land or for which quadrupedalism is only marginally functional is referred to as an obligate biped. Among extant amniotes, obligate bipedalism is found mainly in humans and birds (Hutchinson & Gatesy, 2001; Schmitt, 2003). Similarly, any animal for which quadrupedalism is the exclusive MOL on land or for which bipedalism is inconvenient is referred to as an obligate quadruped. Most extant mammals are obligate quadrupeds (Feldhamer et al., 2007). Among extant reptiles, the vast majority of squamates, turtles (except marine ones) and crocodiles are obligate quadrupeds (Bels & Russell, 2019). Any animal that does not meet either of the above conditions is termed a facultative quadruped or biped.

But what does facultative mean? What is the degree of quadrupedality in a facultative quadruped? While some recent studies have clarified these questions at more narrow phylogenetic scales (Grinham & Norman, 2020a, 2020b), there are to our knowledge no quantitative studies to clarify this term across a broad sample and the literature lacks ethological case studies with temporal data. Of these ambiguous cases, hopping mammals (e.g. kangaroos) are traditionally and predominantly considered facultative quadrupeds (Russo & Kirk, 2017), while walkers (e.g. the bonobo) are mostly considered facultative bipeds (D'Août et al., 2004). Among reptiles, there are also several cases of facultative bipedalism related to running, such as *Basiliscus* (Bels & Russell, 2019; Clemente & Wu, 2018). Some varanids may also adopt a tripod stance in which the tail rests on the ground as part of defensive displays or during intraspecific confrontations (Schuett et al., 2009). As this is a largely static posture, it is not considered here as a case of bipedalism. In the absence of sufficient quantitative data on the subject to settle the question, such as a percentage of locomotor activity (in time or distance travelled) in the two locomotor modes used by a given taxon, we follow this traditional qualitative, categorical approach which, in our opinion, remains relevant and certainly practical.

### 2.3 Postures

Posture was traditionally subject to a gradist or teleological conception of evolution. In the same way that the vertebrate water‐to‐land transition is often represented in a linear sequence (from the lower ‘fishes’ to the higher mammals, of which humans are the most remarkable representatives, passing through the intermediate stages resembling amphibians and reptiles), the sprawling limbs of amphibians and lizards are still sometimes commonly associated with the most ‘primitive’ stage of evolution of terrestrial organisms in comparison to the parasagittally locomoting erect limbs of mammals, which are considered to be the most accomplished stage. Crocodylians, with their sub‐parasagittally locomoting ‘semi‐erect’ limbs, occupy an intermediate place in this diorama of the evolution of land locomotion (e.g. Charig, 1972).

This sprawling‐to‐erect paradigm, mainstream during the early 20th century (Bakker, 1971; Charig, 1972; Gregory, 1912), is now widely contested. Several studies have for example shown that the 'semi‐erect' posture of crocodylians is actually not intermediate. Indeed, it is now commonly accepted that extant crocodylians are descendants of parasagittally locomoting erect forms (Gatesy, 1991a; Parrish, 1987; Reilly & Elias, 1998). Similarly, other studies addressed mammals, pointing out that parasagittally locomoting erect posture is found only in cursorial and graviportal species, with most mammals exhibiting a sub‐parasagittally locomoting crouched posture (Biewener, 1989; Jenkins, 1971). Only the plesiomorphic nature of the posture of urodeles and most squamates has not been refuted by recent research; even the latter is part of a complex multidimensional continuum (Nyakatura et al., 2019).

**FIGURE 1:**
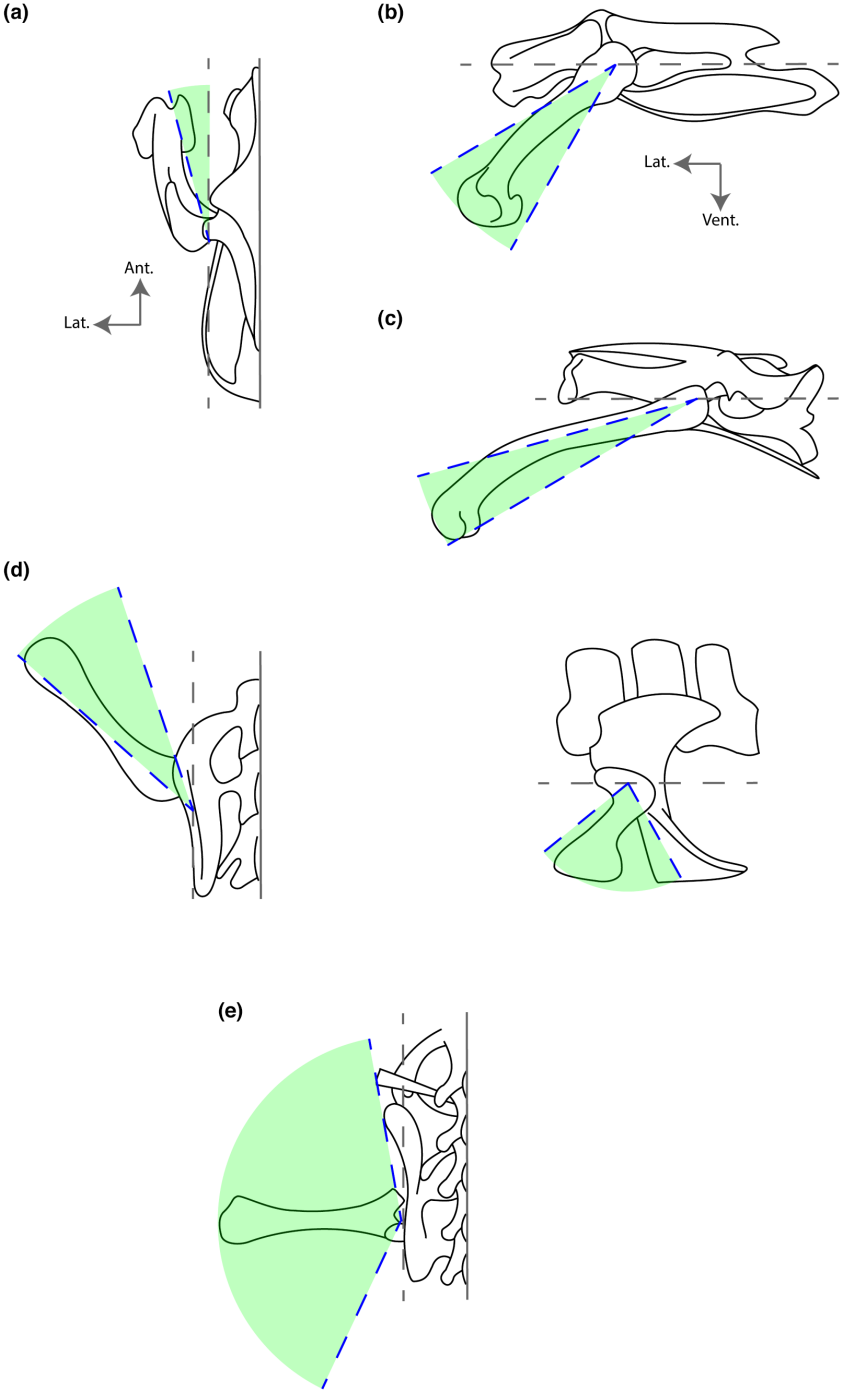
Schematic representation of the pelvic girdle and femur position for different postures (Brinkman, 1981; Carrano, 1998; Galvis et al., 2019; Gatesy, 1991a, 1991b, 1997; Gatesy & Biewener, 1991; Grinham et al., 2019; Jenkins, 1971; Nyakatura et al., 2019; Reilly & Elias, 1998). (a) parasagittal femur in *Rhea Americana* (dorsal view); (b) parasagittally locomoting erect posture in *Rhea Americana* (lateral view); (c) parasagittally locomoting crouched posture in *Columba livia* (lateral view); (d) ‘semi‐erect’ posture in *Alligator mississippiensis* in dorsal (left) and lateral (right) view; (e) sprawling posture in *Iguana iguana* (dorsal view). The grey dotted line represents the parasagittal plane in dorsal view and the dorsal (horizontal) plane in lateral view. The green area represents the total amplitude of femoral excursion, with the blue dotted lines indicating minimum and maximum excursions.

For the purpose of this study, in a context where quantitative data are scarce, we provide the following definitions (Figure 1) based on the literature; mainly based upon normal walking kinematics (Bels & Russell, 2019; Biewener, 1989; Brinkman, 1981; Carrano, 1998; Galvis et al., 2019; Gatesy, 1991a, 1991b, 1997; Gatesy & Biewener, 1991; Grinham et al., 2019; Jenkins, 1971; Nyakatura et al., 2019; Reilly & Elias, 1998):

#### 2.3.1 Parasagittal gait

The femur forms an angle of 15 degrees or less with the parasagittal plane, resulting in a slightly abducted femur. Species with mostly parasagittally moving limbs can present either a more crouched (small birds) or vertically oriented femur (larger birds and cursorial/graviportal mammals).

Crouched—The femur is constrained in a sub‐horizontal position in small birds, between about 15 and 30 degrees below the dorsal horizontal plane.

Erect—The femur of large birds tends to be more upright compared to small species, although it is more horizontal than that of cursorial/graviportal mammals and even non‐avian theropods. Thus, the femur is retracted from the dorsal horizontal plane by about 30–60 degrees in erect birds and by about 35–90 degrees (vertical) in erect mammals, resulting in a more or less antero‐ventral swing of the femur relative to the pelvic girdle.

#### 2.3.2 Sub‐parasagittal gait

Small mammals and, to some respect considering their evolutionary history, crocodylians, can be considered to have a sub‐parasagittal gait. The femur is more abducted compared to fully parasagittally locomoting species, operating between 10 and 30 degrees from the parasagittal plane in small mammals and between 20 and 50 degrees in crocodylians. Sub‐parasagittally locomoting taxa exhibit either a more crouched pose (small mammals) or semi‐vertical femur (crocodylians).

Crouched—The femur in small mammals sometimes protracts slightly above the horizontal plane and regularly retracts beyond the vertical (90 degrees).

Semi‐erect—The femur of crocodylians can be described as held in a 'semi‐erect' posture, although the term is subjective and not entirely appropriate (Gatesy, 1991a ). We use this term to emphasise how the gait of some animals such as Crocodylia tends to be ‘intermediate’ when compared to fully sprawling (e.g. most salamanders) and quite erect (e.g. large mammals) taxa (see Nyakatura et al., 2019).

#### 2.3.3 Sprawling gait

The femur protracts and retracts at an angle of between 15 and 155 degrees to the parasagittal plane and is mainly contained in the dorsal horizontal plane. Lizards and non‐marine turtles are sprawlers.

The MOL and posture assigned to the species in this study, including extinct ones, are available in Table 1 and Supporting Information.

### 2.4 Data acquisition

We measured different geometric and microanatomical parameters on femoral shaft cross‐sections. To do so, we relied mainly on CT scan data that we retrieved from the literature and from MorphoSource. We scanned some of the specimens on the tomography platforms of the Muséum national d'histoire naturelle and the Université de Montpellier. We also used unpublished images of histological sections from the study by Quemeneur et al. (2013). In the literature, the reference plane for the sections is traditionally located at the midpoint of the bone or at the level of the ossification centre (Amson & Kolb, 2016; Houssaye et al., 2018; Quemeneur et al., 2013). For CT scan data, we decided to locate the reference plane where the perimeter of the shaft is the smallest, because this is an area where mechanical stresses are important (Beck et al., 1996; Campione & Evans, 2012; Tommasini et al., 2005). For histological sections, the reference plane is located at the midpoint of the bone. Mixing sections with slightly different reference planes in comparative studies is not considered a problem, however, as long as the species in question do not show excessive microanatomical variation along the shaft (Amson & Kolb, 2016; Houssaye et al., 2018). The scans were processed in ImageJ (Abràmoff et al., 2004) and MorphoDig (Lebrun, 2018). Each bone was oriented so that the section plane was as perpendicular as possible to the axis of the shaft. Data for all left femora were symmetrised so that the sample consisted of right femora only. Some scans were of modest quality, so we increased their resolution by using a bicubic interpolation in ImageJ. Finally, we performed thresholding before taking our microanatomical measurements in ImageJ with the BoneJ plugin (Doube et al., 2010) and BoneProfileR (Girondot & Laurin, 2003; Gônet et al., 2022).

### 2.5 Geometric and microanatomical parameters

We measured various parameters that have previously been associated in the relevant literature with locomotor mode and posture, and more generally with lifestyle in amniotes (Amson & Kolb, 2016; Canoville & Laurin, 2009, 2010; Houssaye & Botton‐Divet, 2018; Houssaye, Waskow, et al., 2016).

**FIGURE 2:**
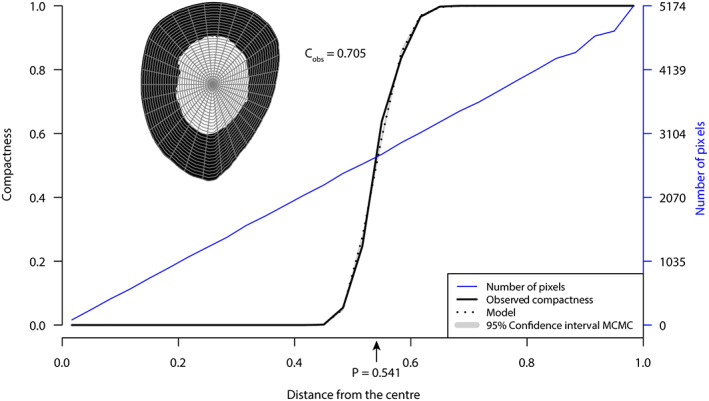
Compactness profile of a femoral diaphyseal thin section of *Allosaurus fragilis* (DNM 2560) as obtained with BoneProfileR (Gônet et al., 2022). The compactness is null in the centre of the section (medulla) and maximum on the edge (cortex). The point P is located at the inflection of the sigmoid curve modelled from the observed compactness; it corresponds to the distance from the centre of the medullocortical transition. In this example, the section is segmented into 30 concentric circles and radii for better readability.

We used BoneProfileR to measure seven compactness parameters. BoneProfileR determines the position of the centre of unmineralisation, that is the centre of the medulla, and segments the cross‐section into 100 concentric circles. The bone compactness (measured by number of bone pixels relative to the total number of pixels) is measured in each of these circles from the centre of the medulla to the edge of the cross‐section. It is between 0 and 1. A compactness of 0 corresponds to an area devoid of bone, while a compactness of 1 corresponds to a surface entirely filled with bone. We can then model a sigmoid curve called compactness profile (Figure 2). From this profile, we extracted the parameter P which corresponds to the inflection point of the sigmoid curve and represents the distance from the centre of the transition between the medulla and the cortex. The reciprocal of the slope of the tangent at point P, called S, reflects the nature of this transition. A high S corresponds to a progressive transition, as in the case of cancellous bone, while a low S reflects an abrupt transition. BoneProfileR also calculates an observed global compactness value, C_obs_. BoneProfileR can also perform radial analyses. The cross‐section is segmented in 60 slices of 6 degrees. Compactness is measured in the same way as explained above and a compactness profile is modelled for each of these slices. An average radial P (RP) and an average radial S (RS) are calculated from the 60 radial P and S values. RSSP and RSSD represent the standard deviation associated with RP and RS and quantify the variability of the medullocortical transition zone, that is its distance from the centre (RSSP) and extent (RSSD) depending on the location on the section. When a species in our sample was represented by more than one individual, the values of these microanatomical parameters were averaged.

**FIGURE 3:**
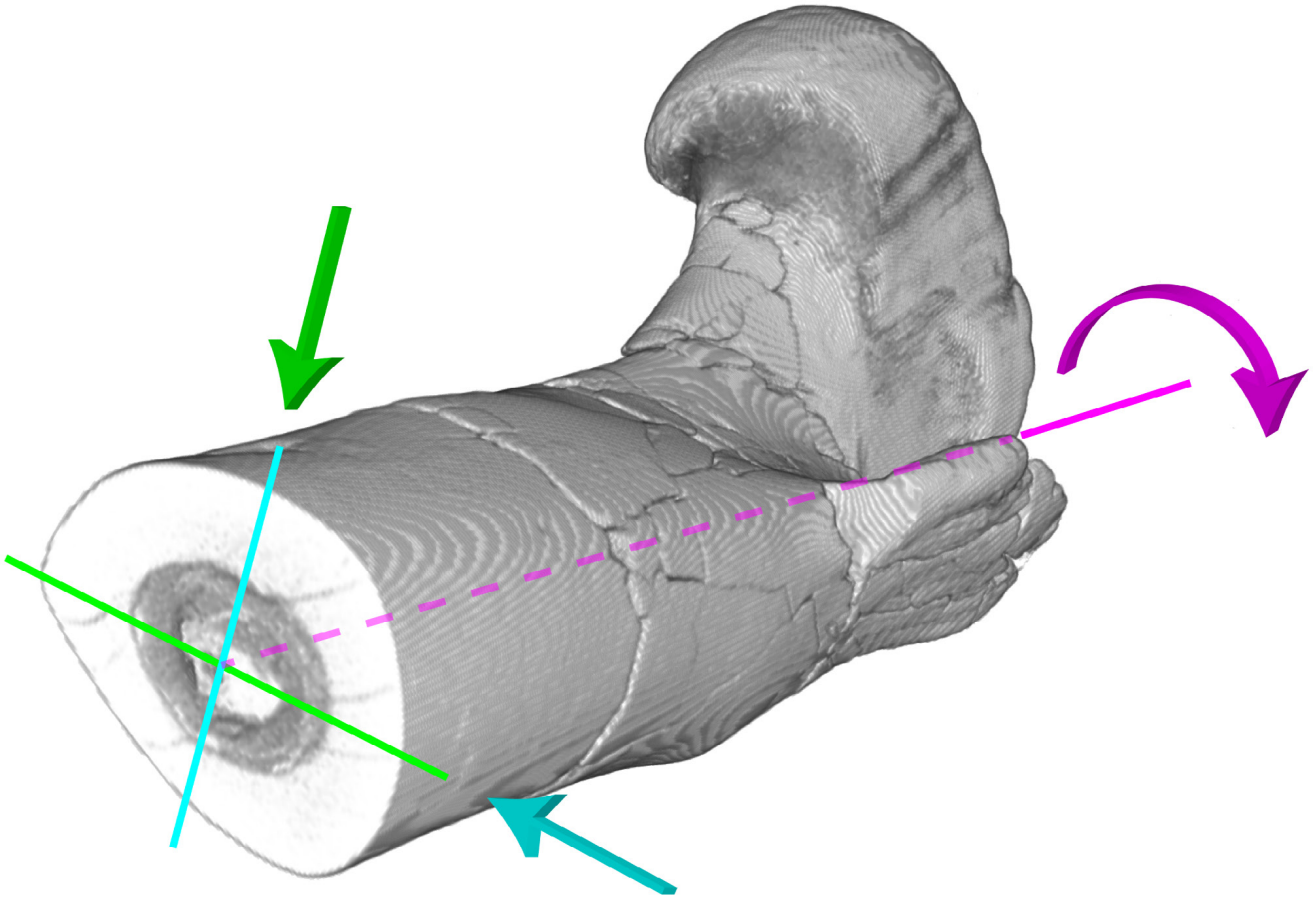
Representation of the different moments of inertia on a proximal end of a femur of *Allosaurus fragilis* (DNM 2560). The area moment of inertia is the greatest around the major axis (blue; I_max_) and reflects a greater resistance to bending against stress perpendicular to this axis (blue arrow). Conversely, the area moment of inertia is the smallest around the minor axis (green; I_min_) and reflects a lower resistance to bending against stress perpendicular to this axis (green arrow). The polar section modulus is calculated around a third axis (purple; Z_pol_) perpendicular to the section plane and passing through the intersection of the major and minor axes. It gives an indication of the resistance to torsion around this axis (purple arrow).

We measured six geometric parameters with BoneJ: Pe_min_, the minimal diaphysis perimeter; BCSA, the area occupied by bone on the section; TCSA, the total area of the section; Ecc, the eccentricity of the section corresponding to the ratio of the area moment of inertia around the major axis (I_max_) to the area moment of inertia around the minor axis (I_min_). The area moment of inertia, or second moment of area, corresponds to the way in which the constituent material of a section is distributed around an axis of reference. It reflects the capacity to resist bending. The higher the area moment of inertia, the greater the resistance to bending. The major and minor axes are perpendicular (Figure 3). They correspond to the axes around which the area moment of inertia is respectively maximum and minimum. In our case, a high ratio value reflects an eccentricity of the bone section. Z_pol_ is the polar section modulus. It corresponds to the area moment of inertia around the axis perpendicular to the plane of the section and passing through the intersection of the major and minor axes (Figure 3). It describes the resistance to torsion of a cylindrical object. In our case, the higher the Z_pol_, the more resistant the bone will be to torsion. SR is the slenderness ratio (1). It corresponds to the ratio of the length of the bone to the square root of the ratio of the area moment of inertia around the minor axis to the total area of the section.

(1) $$\text{Slenderness ratio}=\frac{\text{Bone length}}{\sqrt{\frac{I_{\min}}{\text{TCSA}}}}.$$

A high slenderness ratio SR indicates a slender bone, while a low SR indicates a more robust bone.

### 2.6 Building reference phylogenies

**FIGURE 4:**
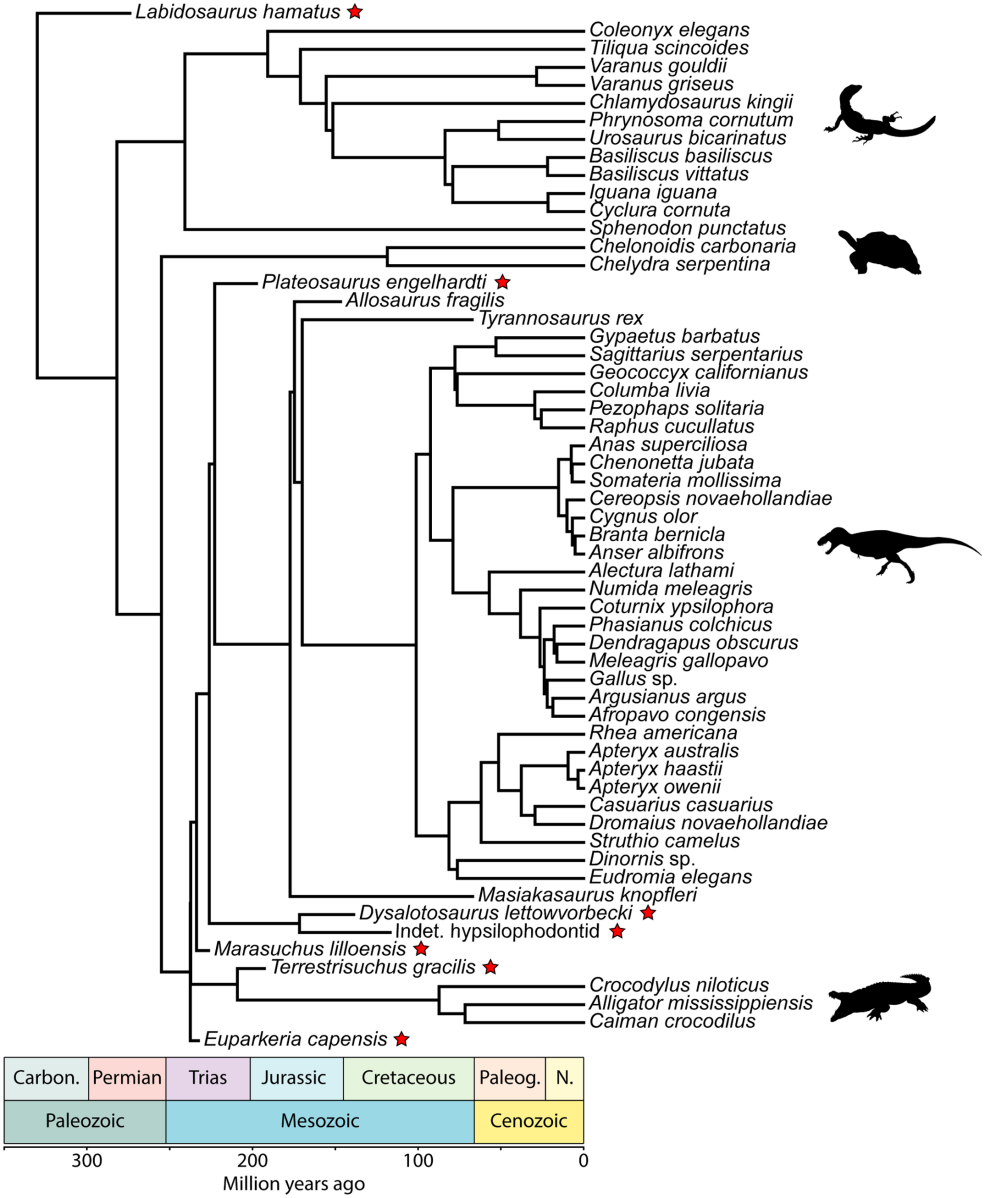
Time‐calibrated phylogenetic tree with the extant and extinct species included in this study. The tree presented here is the first of the set of 100 trees we used for our statistical analyses. The stars indicate the species for which we inferred the mode of locomotion and the posture. The silhouettes come from PhyloPic.

In order to perform phylogenetically informed statistical analyses, we needed a reference phylogeny of amniotes including all the species studied. Such a phylogeny was not available in the literature, so we built a composite phylogeny (Figure 4). Furthermore, we constructed a set of 100 reference trees to include phylogenetic uncertainty in our analyses. However, some portions did not vary because tree sets were not available for all clades (Testudines and Crocodyliformes). The position of the taxa for which we infer the MOL and posture did not vary either. We built the trees in R (R Core Team, 2013) using the packages paleotree (Bapst, 2012), phytools (Revell, 2012) and TreePar (Stadler, 2011). The trees in Newick tree format are available as Supporting Information.

The divergence between Lepidosauria and Archelosauria (Archosauria + Testudines) was set at 281.88 Ma following Turner et al. (2017). Concerning the relationships within lepidosaurs, we extracted on Vertlife a set of 100 trees with only the species of interest from the work of Tonini et al. (2016). Although controversial (Laurin & Piñeiro, 2017; Lichtig & Lucas, 2021), most recent molecular work places turtles as a sister group to archosaurs (Chiari et al., 2012; Irisarri et al., 2017). We follow the latter and set the divergences between turtles and archosaurs and between *Chelydra* and *Chelonoidis* (Durocryptodira) at 255 and 118.57 Ma, respectively, based on Chiari et al. (2012) and Joyce et al. (2013). We set the divergence between Crocodyliformes and Dinosauria at 237.36 Ma based on the time‐calibrated Bayesian tree of archosaurs (vartime = 1) in Turner et al. (2017) and we retrieved the time‐calibrated tree of Crocodyliformes from Drumheller and Wilberg (2020). We used the cal3TimePaleoPhy function in the paleotree package to generate 100 time‐calibrated trees of non‐avian theropods with the tree topology and occurrence matrix of Rauhut & Pol (2019), retrieving speciation, extinction and sampling rates from Bapst et al. (2016). For birds, we extracted 100 trees from the Hackett all species subset on Vertlife (Jetz et al., 2012) and branched them as the sister taxon of *Tyrannosaurus rex* in our non‐avian theropod trees. We used the divergence between *Columba livia* and *Caloenas nicobarica* in our bird trees to attach the *Raphus*‐*Pezophaps* clade. We set the age of divergence between *Raphus cucullatus* and *Pezophaps solitaria* at 25.6 million years following Shapiro et al. (2002). We branched *Dinornis* midway between the Palaeognathae and Tinamidae (*Tinamus major* + *Eudromia elegans*) nodes for each tree. We then removed *Tinamus major* from the trees as it was not part of our sample of reptile femoral cross‐sections initially.

For the statistical analyses, we also needed reference trees that included the taxa for which we wanted to infer posture. We set the divergence between the captorhinid *Labidosaurus* and Diapsida at 323 Ma based on Didier & Laurin (2020). We branched the Triassic archosauriform *Euparkeria*, the dinosauriform *Marasuchus* and the crocodylomorph *Terrestrisuchus* at respectively 237.53, 233.81 and 194.4 Ma following Turner et al. (2017). The divergences between saurischian and ornithischian dinosaurs and between *Plateosaurus* and theropod dinosaurs were set at 226.1 and 222.83 Ma respectively following Turner et al. (2017). Concerning the relationship between ornithopods, we used the consensus tree from Strickson et al. (2016). We considered the hypsilophodont in our sample as a member of clade Elasmaria based on Herne et al. (2019) and inserted it between *Jeholosaurus* and *Hypsilophodon*. The Wonthaggi Formation encompassing the Flat Rocks locality where the hypsilophodont was recovered (Woodward et al., 2018) is late Barremian (~125–127.2 Ma) in age (Herne et al., 2019). We replaced the dryosaurid taxa in Strickson's topology with *Dysalotosaurus*. Its first and last appearance data (FAD and LAD) were considered to be the lower and upper Kimmeridgian boundaries (155.7–150.8), the estimated age for the Middle Dinosaur member of the Tendaguru Formation that yielded remains of this taxon (Sames, 2008). We then used the cal3TimePaleoPhy function of the R package paleotree to calibrate this topology over time based on the FADs and LADs mentioned above.

### 2.7 Phylogenetic flexible discriminant analyses

We imported our reptile dataset into R and log transformed the variables, with the exception of ratios (Ecc and SR). To avoid future complications related to the existence of singular variance–covariance matrices, we generated a dissimilarity matrix for each dataset from the correlation coefficients of the microanatomical variables before performing a hierarchical cluster analysis to identify and eliminate highly correlated variables (correlation coefficient above 0.95). 9 variables out of 13 were retained for our dataset (Pe_min_, Ecc, SR, C_obs_, P, S, RS, RPSD, RSSD).

Linear discriminant analysis (LDA), classically used to separate groups from explanatory variables and to make predictions, cannot remove phylogenetic bias (Motani & Schmitz, 2011). We thus used phylogenetic flexible discriminant analysis (PFDA) devised by Motani & Schmitz (2011). It is derived from flexible discriminant analysis (FDA; Hastie et al., 1994) and corresponds to its phylogenetically informed version. FDA is a classification model based on a combination of linear regression models. It employs optimal scoring to transform the response variable. To quote Montani & Schmitz: ‘Given that FDA reduces a discrimination problem to a regression problem, it is compatible with the common framework of phylogenetic bias removal, which is a GLS problem’. In practice, PFDA incorporates a phylogenetic distance matrix whose terms are multiplied by lambda (Pagel, 1999). Lambda is optimised to minimise the model error, that is the part of variance explained by the phylogeny. Its value is comprised between 0 and 1. A lambda of 0 indicates that the phylogeny does not explain trait distribution, while a lambda of 1 indicates that the phylogeny explains as much variance in the character as under a Brownian model of evolution. In other words, PFDA is used to explain a factor (in our case, locomotor mode and posture) from explanatory variables (here, geometric and microanatomical parameters) while taking phylogeny into account. PFDAs were performed with all of our 100 trees since a small variation in lambda can impact the results. For a detailed description of PFDA, refer to Motani & Schmitz (2011). The PFDA R script is available as Supporting Information.

### 2.8 Overfitting and taphonomic aspect

Overfitting occurs when a model becomes too complex, that is, when the number of its estimated parameters becomes too high (Everitt & Skrondal, 2010). An overfitted model will perform very well in explaining the initial data (training) while performing poorly with new data or predictions (testing). The key to selecting the best model lies in optimising the choice of parameters to be included to minimise test errors. In our case, the selection criterion we used is the percentage of correct classification (PCC) obtained after a cross‐validation procedure (CV; Stone, 1974). The latter consists of: (1) extracting a species among the n species of a training data set, (2) generating a model with n−1 observations, (3) predicting the posture of the extracted species. The operation is repeated for each of the n species in the training data set, with replacement in each round. Since the posture of the species in the training set is known, it is possible to produce a PCC. The higher this percentage, the better the model performs under the test conditions. Cross‐validation was performed for all possible combinations of parameters and for each of the 100 phylogenetic trees, for a total of more than 50,000 cross‐validation procedures. We averaged the PCC for each combination. The Akaike information criterion (AIC; Akaike, 1974) was also calculated for each model. In the end, we retained two models which are presented in Table 3.

**TABLE 3:** List of the models retained after the cross‐validation procedures.

Model	Trait	Mean PCC (%)	Variables retained	Mean AIC
Reptile 1	Locomotor mode	82	C_obs_; P; RS; Pe_min_	0.979
Reptile 2	Posture	66	P; RSSD; Pe_min_; SR; Ecc	0.562
Reptile 3	Posture	60	C_obs_; RS; Pe_min_; SR	0.648

*Note*: PCC and AIC are averaged over the 100 time‐calibrated phylogenetic trees.

Abbreviations: AIC, Akaike information criterion (Akaike, 1974); PCC, percentage of correct classification.

Among the parameters retained for our second model is the eccentricity of the cross‐section. The latter can be strongly impacted by taphonomic processes in the case of fossils, which can be problematic in an inferential framework. This is why we selected a third model for which the Ecc parameter was deliberately discarded (Table 3).

### 2.9 Numerical description of the cross‐sections

**FIGURE 5:**
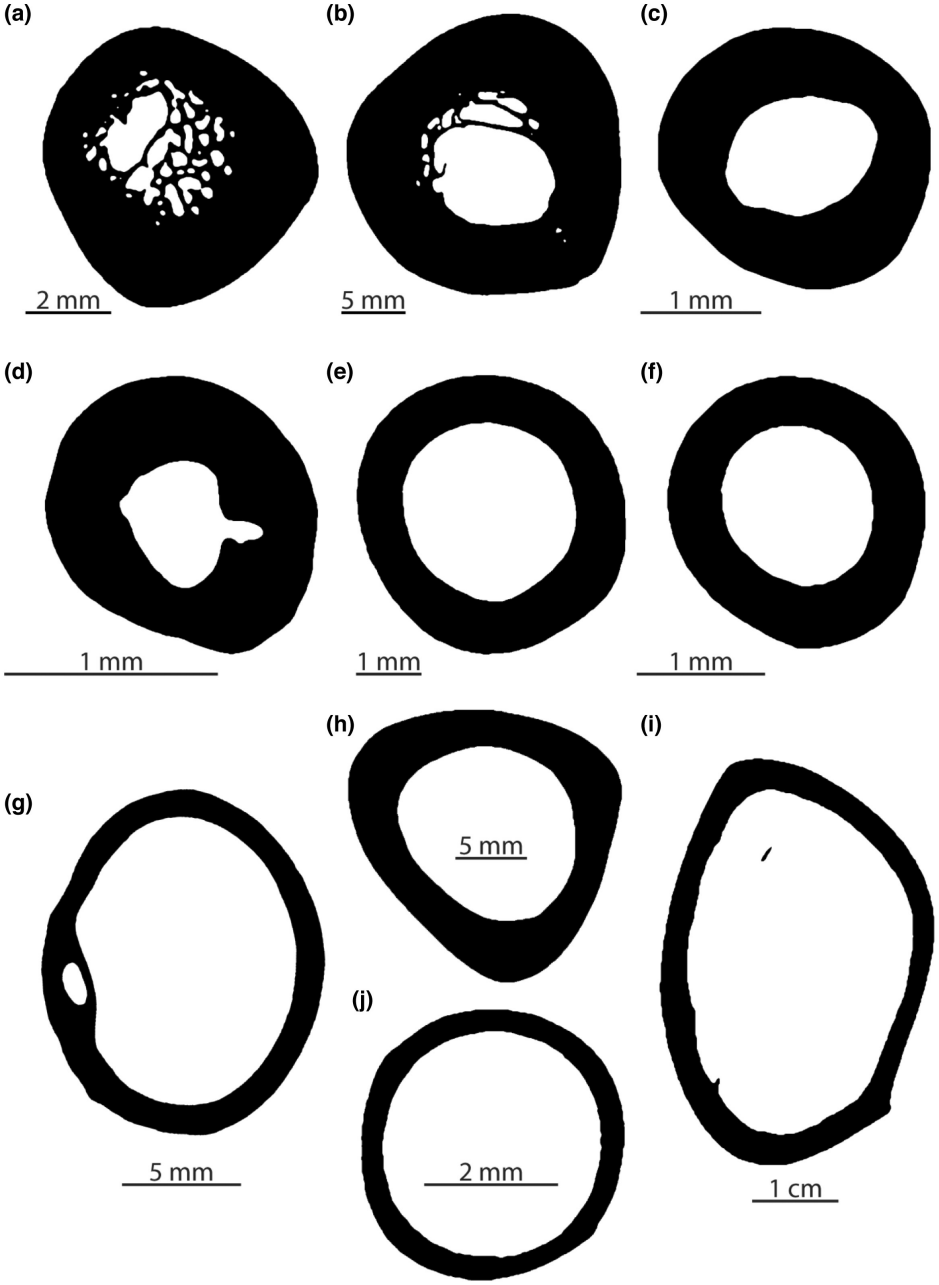
Some of the femoral thin sections used in this study. (a) *Chelonoidis carbonaria* MNHN‐ZA‐AC‐1877‐404 (quadruped, sprawling); (b) *Crocodylus niloticus* MNHN‐ZA‐AC‐1963‐22 (quadruped, ‘semi‐erect’); (c) *Tiliqua scindoides* MNHN‐ZA‐AC‐1898‐285 (quadruped, sprawling); (d), *Phrynosoma cornutum* MNHN‐ZA‐AC‐1893‐662 (quadruped, sprawling); (e), *Chlamydosaurus kingii* ypm:vz:ypm herr 010336 (facultative biped, sprawling); (f) *Basiliscus basiliscus* MNHN‐ZA‐AC‐1888‐124 (facultative biped, sprawling); (g) *Gypaetus barbatus* MNHN‐ZO‐AC‐1993‐52 (biped, parasagittally locomoting crouched); (h) *Masiakasaurus knopfleri* FMNH PR 2117 (biped, erect); (i) *Struthio camelus* YPM 2124 (biped, erect); (j) *Columba livia* RVC (biped, parasagittally locomoting crouched).

A sample of the thin sections used in this study is presented in Figure 5. We performed phylogenetic analyses of variance (ANOVA) in R using the geiger (Pennell et al., 2014) and phytools (Revell, 2012) packages to investigate differences between locomotor modes and postures in the geometric and microanatomical parameters underlying our models. When an ANOVA was significant, we performed pairwise post hoc tests with the Bonferroni correction to explore the differences between group means while controlling for the experimental error rate. ANOVA is fairly robust to data departures from normality (Glass et al., 1972; Lix et al., 1996). It is less robust to violations of the equality of variance assumption, especially when group sizes are unequal (Keppel, 1991). Our data sometimes violated the latter assumption. To overcome this issue, outliers were removed from the data for the relevant parameters: *Varanus gouldii*, *Iguana iguana* and *Allosaurus fragilis* (P/locomotion model); *Iguana iguana*, *Coleonyx elegans*, *Phrynosoma cornutum*, *Allosaurus fragilis*, *Chelonoidis carbonaria* and *Chelydra serpentina* (P/postural model).

### 2.10 Phylogenetic signal

We estimated the phylogenetic signal in locomotor modes and postures and in each geometric and microanatomical parameters retained in our models. For categorical traits (locomotor modes and postures), we used the delta‐statistic (Borges et al., 2019). The delta‐statistic depends on the uncertainty associated with the inference of ancestral states. It is accepted that the more a trait follows the phylogeny, the less uncertainty there is in the reconstruction of ancestral states. The calculation of uncertainty is based on Shannon's concept of entropy (Shannon, 1948). The lower the uncertainty, the lower the entropy and the higher the value of delta. The higher delta is, the stronger the phylogenetic signal. A *p*‐value is obtained through a randomisation process: the states of the trait under consideration are randomly shuffled and a new delta‐statistic is calculated. The operation is repeated 1000 times before comparing the randomised delta‐values to the initial one. For continuous traits (geometric and microanatomical parameters), we used lambda (Pagel, 1999). This was done with the phylosig function of the phytools package in R (Revell, 2012). The function also allows the user to perform a likelihood ratio test to obtain an associated *p*‐value for lambda.

### 2.11 Body mass estimates and phylogenetic generalised least squares

Scaling describes how certain biological traits in individuals vary with body size, which may include allometry or disproportionate change of a trait with size (Gould, 1966). Knowing this, it is important to consider this size effect when studying a biological trait in a large number of individuals that vary greatly in size. For this work, we considered body mass as a proxy for body size. We gathered mass estimates from the literature for each of the species in our sample. For extant species, we mainly relied on the database of Myhrvold et al. (2015) which compiled median mass values for a large proportion of amniote taxa. In some particular cases, when the species is not known, for example *Gallus* sp., we calculated the average mass of the species in the genus. In some cases, the species was absent from the database, and this is especially the case for extinct species. We used the cQE function from the R package MASSTIMATE (Campione, 2020) to estimate the body mass of the bipedal non‐avian dinosaurs in our sample from femoral circumference (Pe_min_). The cQE function employs the equation for bipeds presented in Campione et al. (2014) which is a corrected version of the equation for quadrupeds in Campione and Evans (2012). Preservation of the *Tyrannosaurus* femur (MOR 1125) prevented us from extracting a cross‐section where the perimeter was the smallest, resulting in a likely overestimation of body mass. Therefore, for an estimate of *Tyrannosaurus* body mass, we relied on the literature (Campione et al., 2014; Hutchinson et al., 2011). All mass estimates with their provenance are available in Table 1 and Supplementary Material 1.

We used phylogenetic generalised least squares (PGLS) in R to study a potential influence of body size on the different geometric and microanatomical parameters retained in our models. We performed PGLS using the caper package (Orme et al., 2018). PGLS fits a linear regression between a dependent variable and one or more independent variables while accounting for phylogeny (Symonds & Blomberg, 2014). This is done by adjusting branch length transformations with lambda (Pagel, 1999). PGLS were performed with 100 phylogenetic trees.

### 2.12 Impact of functional ecology and body mass on the model axes

In order to investigate a possible effect of lifestyle on locomotor mode and posture, and given that several environments may involve the same functional demand (Bels & Russell, 2019), we defined four ecological categories based on limb use in relation to the known or inferred environment: (1) semi‐aquatic, which refers to an animal adapted to both terrestrial and aquatic environments, between which it shares its time. In the water, semiaquatic animals move by swimming (thrust is generated by undulatory movements of all or part of the body and/or by movements of the limbs) or by walking on the bottom (Dunstone & Gorman, 2007). (2) terrestrial, referring to an animal that spends all its time on the ground (ground dwellers). (3) fossorial, referring to an animal that spends most of its time underground, including for foraging, or that habitually retreats into a burrow‐type underground shelter excavated by itself for protective and/or thermoregulatory purposes, even though digging may represent only a small proportion of its activity. An animal that anecdotally digs into the substrate to hibernate/aestivate and/or lay its eggs is not considered as such. Fossorial species present adaptations related to digging, such as robust forelimbs and long clawed fingers (Shimer, 1903). Such species tend to use their forelimbs for excavation and hind limbs to kick the loosened soil backward (Reichman & Smith, 1990; Thompson, 1995) (4) arboreal, referring to an animal that spends a significant amount of time climbing trees or inclined surfaces, although it can be engaged in various activities on the ground, such as foraging. Elongated, slender forelimbs and clawed fingers, among other things, are often found in arboreal species (Dublin, 1903); also termed scansorial. The functional ecology attributed to the species studied here, including extinct ones, are listed in Table 1 and Supplementary Material 1.

We designed several linear models in R to compare the means of the coordinates of our extant species on the first two discriminant axes generated by the PFDAs across functional ecology categories while controlling for the effect of body mass. First, we added a constant value equal to the lowest coordinate for each of the first two axes of our models in order to transform negative coordinates into positive ones. We then log_10_ transformed our coordinates. In total, we developed four linear models. We incorporated the interaction term between functional ecology and body mass when it was significant. When there was a significant difference between functional ecologies for a given parameter, we performed pairwise post hoc tests with false rate discovery (FDR) corrections.

Also, to better assess the impact of body mass on our PFDA models, we removed from the models in question all the parameters that were significantly associated with body mass. We performed a series of cross‐validation procedures and compared the results to the original models to evaluate the contribution of body mass to classification performance.

### 2.13 Phylogenetic nomenclature

The terms reptile and Reptilia is used throughout the article following the definition of Laurin & Reisz (2020): ‘The smallest crown clade containing *Testudo graeca* Linnaeus 1758 (*Testudines*), *Iguana iguana* Linnaeus 1758 (*Lepidosauria*), and *Crocodylus* (originally *Lacerta*) *niloticus* Laurenti 1768 (*Archosauria*)’. Therefore, Reptilia is composed of turtles, lepidosaurs, archosaurs (including birds), and all extinct forms that derive from their most recent common ancestors.

### 2.14 Institutional abbreviations

CM, Carnegie Museum of Natural History, Pittsburgh, Pennsylvania, USA; DNM, Natural History Museum of Utah, Salt Lake City, Utah, USA; FMNH, Field Museum of Natural History, Chicago, Illinois, USA; GPIT, Geologisch‐Paläontologisches Institut, Tübingen, DE; MNHN, Muséum national d'histoire naturelle, Paris, FR; MOR, Museum of the Rockies, Bozeman, Montana, USA; NHMUK, Natural History Museum, London, UK; NMV, Museums Victoria, Melbourne, AU; PJB, Peter J. Bishop personal collection; PVL, Instituto Miguel Lillo, Tucumán, AR; RVC, Royal Veterinary College, London, UK; SAM PK, Iziko South African Museum, Cape Town, ZA; SMNS, Staatliches Museum für Naturkunde, Stuttgart, DE; UMZC, Cambridge University Museum of Zoology, Cambridge, UK; YPM, Yale Peabody Museum of Natural History, New Haven, Connecticut, USA.

## 3 RESULTS

### 3.1 Characterisation of the cross‐sections

Based on the results of the phylogenetic ANOVAs, among all parameters, only P (distance from the centre of the section of the medullocortical transition) was significantly different between the locomotor (MOL) and postural groups (0.008 < *p*‐value < 0.034; mean = 0.017 and 0.02 < *p*‐value < 0.047; mean = 0.032, respectively; Table 4). For the locomotion model, after pairwise post hoc tests with *p*‐value corrections (see Supporting Information: Table S1), bipeds had a significantly higher P than quadrupeds (mean = 0.76 and 0.49, respectively). Facultative bipeds had an intermediate mean P (0.61) that was not significantly different from that of bipeds and quadrupeds. For the postural model, pairwise post hoc tests (see Supporting Information: Table S1) showed that parasagittally locomoting crouched taxa always (mean P = 0.79) and erect taxa sometimes (0.012 < *p*‐value < 0.102; mean P = 0.71) were significantly different from 'semi‐erect' taxa (mean P = 0.56) but not from each other and from sprawling taxa (mean P = 0.59).

**TABLE 4:** List of the phylogenetic ANOVAs performed in this study. *p*‐values are averaged over 100 phylogenetic trees.

Phylogenetic ANOVA model formula	Mean *p*‐value
$C_{\mathrm{obs}}∼\text{Locomotor mode}$	0.18
P∼Locomotor mode	0.017*
RS∼Locomotor mode	0.54
${\mathrm{Pe}}_{\min}∼\text{Locomotor mode}$	0.442
Ecc∼Posture	0.45
P∼Posture	0.032*
RSSD∼Posture	0.64
${\mathrm{Pe}}_{\min}∼\text{Posture}$	0.08
SR∼Posture	0.563

* Indicates a mean *p*svalue below 0.05.

### 3.2 Phylogenetic signal

A phylogenetic signal was found for both locomotion and posture (Table 5). Delta ranged from 5.5 to 414.6 (mean = 31.6) for the mode of locomotion and from 4.7 to 14.77 (mean = 10.293) for the posture. These results were consistently significant (*p*‐values < 0.001). A strong phylogenetic signal was found for the geometric parameters Pe_min_ (minimum perimeter of the cross‐section) and SR (slenderness ratio), with lambda being respectively between 0.987 and 0.999 (mean = 0.999) and between 0.774 and 0.889 (mean = 0.826), and a *p*‐value well below 0.001 in each case. The compactness parameters C_obs_ (observed bone compactness) and P also contained a substantial phylogenetic signal between 0.7 and 0.747 (mean = 0.706) and between 0.91 and 0.955 (mean = 0.933), respectively (*p*‐values < 0.001). The RS parameter (radial S; the average of the measurements of S [reciprocal of the asymptote slope at P on the compactness profile] all around the section) appeared to have a weaker signal with a value of lambda between 0.322 and 0.482 (mean = 0.377) and a *p*‐value that was close to significance (0.06–0.13; mean = 0.1). RSSD (standard deviation of RS) and Ecc (cross‐sectional eccentricity) did not contain a phylogenetic signal based on our sample (mean lambda = 0.16 and <0.001, respectively; mean *p*‐value = 0.28 and 1, respectively).

**TABLE 5:** Phylogenetic signal in the data. Values are averaged over 100 time‐calibrated trees.

Trait/Parameter	Mean delta	Mean lambda	Mean *p*‐value
Locomotor mode	31.6		<0.001***
Posture	10.293		<0.001***
C_obs_		0.706	<0.001***
P		0.933	<0.001***
RS		0.377	0.1
RSSD		0.16	0.28
Pe_min_		0.999	<0.001***
SR		0.826	<0.001***
Ecc		<0.001	1

*** Indicates a mean *p*‐value below 0.001.

### 3.3 Effect of body mass on the variables in the models

Body mass was significantly associated with Pe_min_ (*p*‐values < 0.001), SR (*p*‐values < 0.001) and Ecc (0.001 < *p*‐value < 0.001; see Table 6). The lambda parameter for Pe_min_ and SR ranged from 0.515 to 0.586 (mean = 0.539) and from 0.689 to 0.826 (mean = 0.763), respectively. Lambda was always below 0.001 for Ecc. However, no association was found between body mass and C_obs_ (0.262 < *p*‐value < 0.416; mean = 0.326), P (0.375 < *p*‐value < 0.748; mean = 0.53), RS (0.075 < *p*‐value < 0.157; mean = 0.109) and RSSD (0.185 < *p*‐value < 0.282; mean = 0.229).

**TABLE 6:** Relationship between the femoral geometric and microanatomical parameters retained in our models and body mass. Values reported are means obtained from 100 phylogenetic trees.

PGLS model formula	*R* ^2^	*p*‐value	Lambda
$C_{\mathrm{obs}}\sim \text{Body mass}$	0.02	0.326	0.704
P~Body mass	0.009	0.53	0.937
RS~Body mass	0.052	0.109	0.572
${\mathrm{Pe}}_{\min}\sim \text{Body mass}$	0.927	<0.001***	0.539
RSSD~Body mass	0.03	0.229	0.272
SR~Body mass	0.248	<0.001***	0.763
Ecc~Body mass	0.191	0.001**	<0.001

**,*** Indicates a mean *p*‐value below 0.01 and 0.001.

### 3.4 Morphometric separation of locomotor and postural groups

**FIGURE 6:**
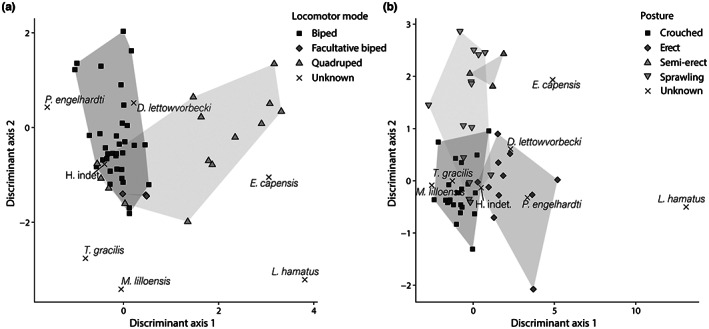
Morphometric separation of reptiles according to their locomotion and posture, as shown by phylogenetic discriminant spaces generated by PFDA on femoral geometric and microanatomical data with tree 1 (varanids and iguanids included). (a) model 1 for the trait locomotor mode. Inferences: biped, *Plateosaurus engelhardti*, the indeterminate hypsilophodontid, *Dysalotosaurus lettowvorbecki*; facultative biped, *Terrestrisuchus gracilis*, *Marasuchus lilloensis*; quadruped, *Euparkeria capensis*, *Labidosaurus hamatus*. (b) model 2 for the trait posture. Inferences: sprawling, *Marasuchus lilloensis*, *Terrestrisuchus gracilis*, the indeterminate hypsilophodontid; erect, *Dysalotosaurus lettowvorbecki*, *Plateosaurus engelhardti*, *Euparkeria capensis*, *Labidosaurus hamatus*.

The models generated from our geometric and microanatomical measurements on the femur successfully discriminated locomotor and postural groups. Figure 6a shows that the phylogenetic discriminant space produced from the axes derived from PFDA with tree 1 discriminates reasonably well bipeds from quadrupeds, but occasional bipeds overlap both other categories. The variables retained for the analysis are C_obs_, P, RS and Pe_min_ (model 1; see Table 3). The groups correspond to the three levels of the trait locomotor mode (quadruped, biped and facultative biped).

The percentage of correct classification (PCC) for the entire tree population ranged from 84 to 86% (mean = 84%). The 34 bipeds in the sample were classified correctly in almost all cases (97%–100% of correct classification; mean = 97%). The 14 quadrupeds were also classified correctly in the majority of cases (71% of correct classification). The misclassified taxa, the varanids *Varanus gouldii* and *Varanus griseus*, and the iguanids *Iguana iguana* and *Cyclura cornuta*, were found among bipeds. Indeed, they were at the origin of the overlap between bipeds and quadrupeds on the graph. When they were removed from the analysis, the average PCC reached 94% and increased from 82 to 91% in CV (cross‐validation). When removing varanids and iguanids, all bipeds and quadrupeds were correctly classified for all trees (see Supporting Information: Figure S1). The 3 facultative bipeds were systematically misclassified with or without varanids and iguanids in the sample. When the varanids and iguanids were included, in the majority of the phylogenetic hypotheses, 2/3 of the facultative bipeds were found in bipeds, 1/3 in quadrupeds. When the varanids and iguanids were excluded from the analysis, the facultative bipeds were always found among the bipeds.

The inferences made for the taxa of interest were generally plausible and consistent. The captorhinid *Labidosaurus* and the archosauriform *Euparkeria* were always inferred as quadrupeds. The dinosauriform *Marasuchus* is inferred as a facultative biped. The dinosaurs *Plateosaurus*, *Dysalotosaurus* and the indeterminate hypsilophodont were inferred bipeds. The crocodylomorph *Terrestrisuchus* was inferred as a biped in 70% of the tree hypotheses. The rest of the time, it was inferred as a facultative biped. When removing varanids and iguanids from the analysis, *Terrestrisuchus* was inferred as a biped in 100% of cases (see Supporting Information: Figure S1).

Lambda ranged between 0.3 and 0.36 over the entire tree population (mean = 0.33). However, the results obtained varied little between these two extremes and the inferences for the taxa of interest were the same except for *Terrestrisuchus gracilis* (see above).

Figure 6b shows a moderately good separation of postural categories according to the phylogenetic discriminant space produced from the axes derived from PFDA with tree 1. The variables retained for the analysis were P, RSSD, Pe_min_, SR and Ecc (model 2; see Table 3). The groups correspond to the four levels of the trait posture (parasagittally locomoting crouched, erect, 'semi‐erect' and sprawling).

The PCC for the entire tree population ranged from 82% to 84% (mean = 82%). The 23 crouched species in the sample showed a good classification rate (78%–83% of correct classification; mean = 78%). This was also the case for the 11 erect and 14 sprawling (82% and 93% of correct classification, respectively). The 3 'semi‐erect' species showed 67% of correct classification. However, the presence of varanids and iguanids in our sample affected these results. Again, varanids and iguanids were responsible for the overlap between postural categories. If we removed them from the analysis, crouched species achieved an average of 91% of correct classification, as did erect species; sprawling species reached 100% of correct classification, while 'semi‐erect' species remained at 67%. The PCC reached 91% for this combination of parameters and increased from 66 to 81% in CV (see Supporting Information: Figure S2).

The inferences made for the taxa of interest were sometimes surprising. The captorhinid *Labidosaurus* was always inferred as erect, while *Marasuchus*, *Terrestrisuchus* and the indeterminate hypsilophodontid were inferred as sprawlers; and *Euparkeria* was always inferred as erect. As expected, the dinosaurs *Plateosaurus* and *Dysalotosaurus* were inferred erect in all cases. If varanids and iguanids were removed, the indeterminate hypsilophodontid is assessed as crouched with all tree hypotheses (see Supporting Information: Figure S2). However, taphonomy could also affect these scores. The best model retained without the parameter Ecc (model 3; see Table 3) gave a PCC of 73% (60% in CV), 81% without varanids and iguanids (70% in CV). The new inferences, which seemed mostly an improvement, were as follows: *Euparkeria*, *Terrestrisuchus* and *Marasuchus* were inferred sprawlers; *Labidosaurus* was inferred 'semi‐erect'; *Plateosaurus*, *Dysalotosaurus* and the indeterminate hypsilophodontid were inferred erect, with or without the varanids and iguanids (see Supporting Information: Figure S3).

Based on analyses on the whole tree population, lambda varies between 0.27 and 0.3 (mean = 0.28), which means that the phylogenetic signal was weak. However, the results obtained with the initial model barely varied between these two extremes and the inferences for the taxa of interest were the same.

### 3.5 Locomotor modes and postures: Relationship with functional ecology and body mass

**FIGURE 7:**
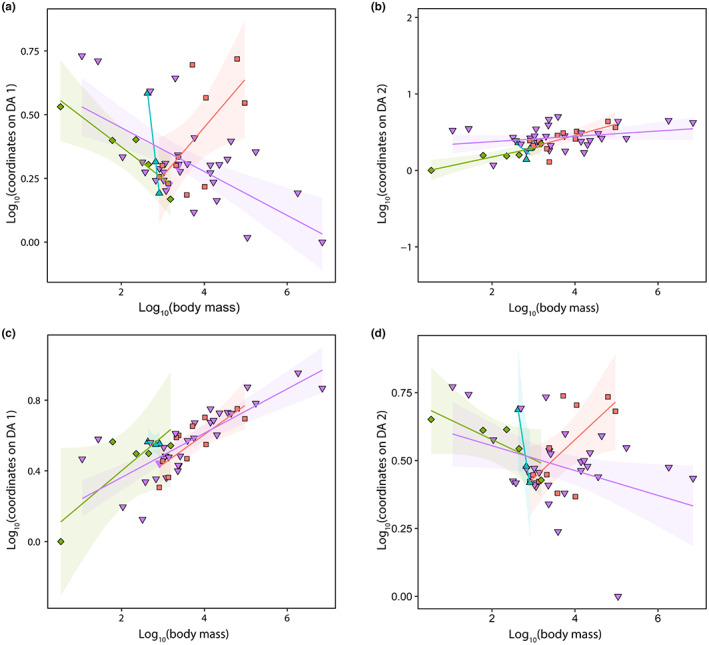
Influence of body mass on the two main axes of our locomotion (a and b) and postural (c and d) models. The green diamonds represent arboreal taxa; the purple triangles, terrestrial species; the blue triangles, fossorial species; the red squares, semi‐aquatic species.

The coordinates on the first axis of the locomotion model were always significantly associated with body mass (*p*‐values < 0.002; mean < 0.001) but never with functional ecology (0.181 < *p*‐value < 0.224; mean = 0.202; Table 7). However, differences between regression slopes for the different locomotor modes were significant (*p*‐values < 0.001; Figure 7a). The coordinates on the second axis were always significantly associated with body mass (0.004 < *p*‐value < 0.008; mean = 0.006) and only once with functional ecology (*p*‐value = 0.048; tree 22). For the other trees, the p‐value approached the significance level without ever reaching it (0.051 < *p*‐value < 0.09; mean = 0.07). The results of the post hoc tests for tree 22 were all non‐significant (see Supporting Information: Table S2).

**TABLE 7:** Effect of functional ecology, body mass and the interaction of the two on the first two axes of our locomotion and postural models. *p*‐values are averaged over 100 phylogenetic trees.

Model	Linear model formula	Term	Mean *p*‐value
Locomotor mode	DA1~Functional ecology*Body mass	Functional ecology	0.202
		Body mass	<0.001***
		Interaction	<0.001***
	DA2~Functional ecology*Body mass	Functional ecology	0.07
		Body mass	0.006**
		Interaction	0.182
Posture	DA1~Functional ecology*Body mass	Functional ecology	0.38
		Body mass	<0.001***
		Interaction	0.543
	DA2~Functional ecology*Body mass	Functional ecology	0.622
		Body mass	0.091
		Interaction	0.015*

Abbreviation: DA, discriminant axis.

* Indicates a mean *p*‐value below 0.05.

**,*** Indicates a mean *p*‐value below 0.01 and 0.001.

The coordinates on the first axis of the postural model were always significantly associated with body mass (*p*‐values < 0.001) and never with functional ecology (0.356 < *p*‐value < 0.404; mean = 0.38). The coordinates on the second axis were almost significantly associated with body mass (0.062 < *p*‐value < 0.116; mean = 0.091), but this relationship never reached the significance level. These coordinates were never correlated with functional ecology (0.603 < *p*‐value < 0.637; mean = 0.622). However, interactions existed between the regression slopes (0.014 < *p*‐value < 0.017; mean = 0.015; Figure 7d).

When we retained only the parameter Pe_min_ correlated with size (see Table 6) in our locomotion model to assess the contribution of body mass to classification performance, the latter decreased by almost half (−36 percentage points of correct classification in cross‐validation compared with the original model; see Table 8). Similarly, when we retained only the parameters Pe_min_, Ecc and SR, and the parameters Pe_min_ and SR in our two postural models including and excluding cross‐sectional eccentricity, respectively, classification performance decreased by almost half (−27 and −23 percentage points of correct classification in cross‐validation, respectively; see Table 8).

**TABLE 8:** Cross‐validation results for the PFDA models including only the body mass correlated parameters.

Model	Trait	Mean PCC (%)	Variables retained
Reptile 1 (BM only)	Locomotor mode	46	Pe_min_
Reptile 2 (BM only)	Posture	39	Pe_min_; SR; Ecc
Reptile 3 (BM only)	Posture	37	Pe_min_; SR

*Note*: PCC is averaged over 100 time‐calibrated phylogenetic trees. Abbreviations: BM, body mass; PCC, percentage of correct classification.

## 4 DISCUSSION

### 4.1 Characterisation of the sections

Bipeds appear to have a higher P (distance from the centre of the section of the medullocortical transition) than quadrupeds, indicating that the medullary region is larger. However, according to our results, femoral compactness at the mid‐diaphysis (C_obs_) does not seem to differ between bipeds and quadrupeds when phylogeny is taken into consideration. This is surprising, as the parameter P and bone compactness generally evolve in an inverse way: as P increases, compactness decreases, and vice versa. This relationship between C_obs_ and P is sometimes referred to in the literature as the corticodiaphyseal index (Canoville & Laurin, 2009; Castanet & Caetano, 1995). In this case, considering P, we would have expected a significantly lower compactness in bipeds. Bone compactness is strongly influenced by the environment, for example high compactness can sometimes be explained by an aquatic lifestyle (pachyostosis and osteosclerosis). Indeed, an increase in compactness reduces buoyancy, so that the animal can remain passively underwater, thus limiting its energetic consumption (Germain & Laurin, 2005; Houssaye, Sander, et al., 2016). Conversely in birds, a decrease in compactness reduces the overall body mass of the animal, which is an advantage for flight (Dumont, 2010). But in our case, our reptile sample does not contain any fully aquatic species and the birds in our sample are mostly ground birds or poor flyers. However, it is true that some of our sampled birds (e.g. ratites) are secondarily flightless. Thus, it is conceivable that they evolved through a flight bottleneck that may have limited the compactness of the femur. The addition of other non‐avian theropods to the sample should provide further insight. Although beyond the scope of this study, this question definitely deserves further investigation. Large body mass (graviportality) can also affect the cortical area (Houssaye, Waskow, et al., 2016). But again, our sample does not contain particularly graviportal taxa, and there is no significant association between body mass and bone compactness with our sample (Table 6).

**FIGURE 8:**
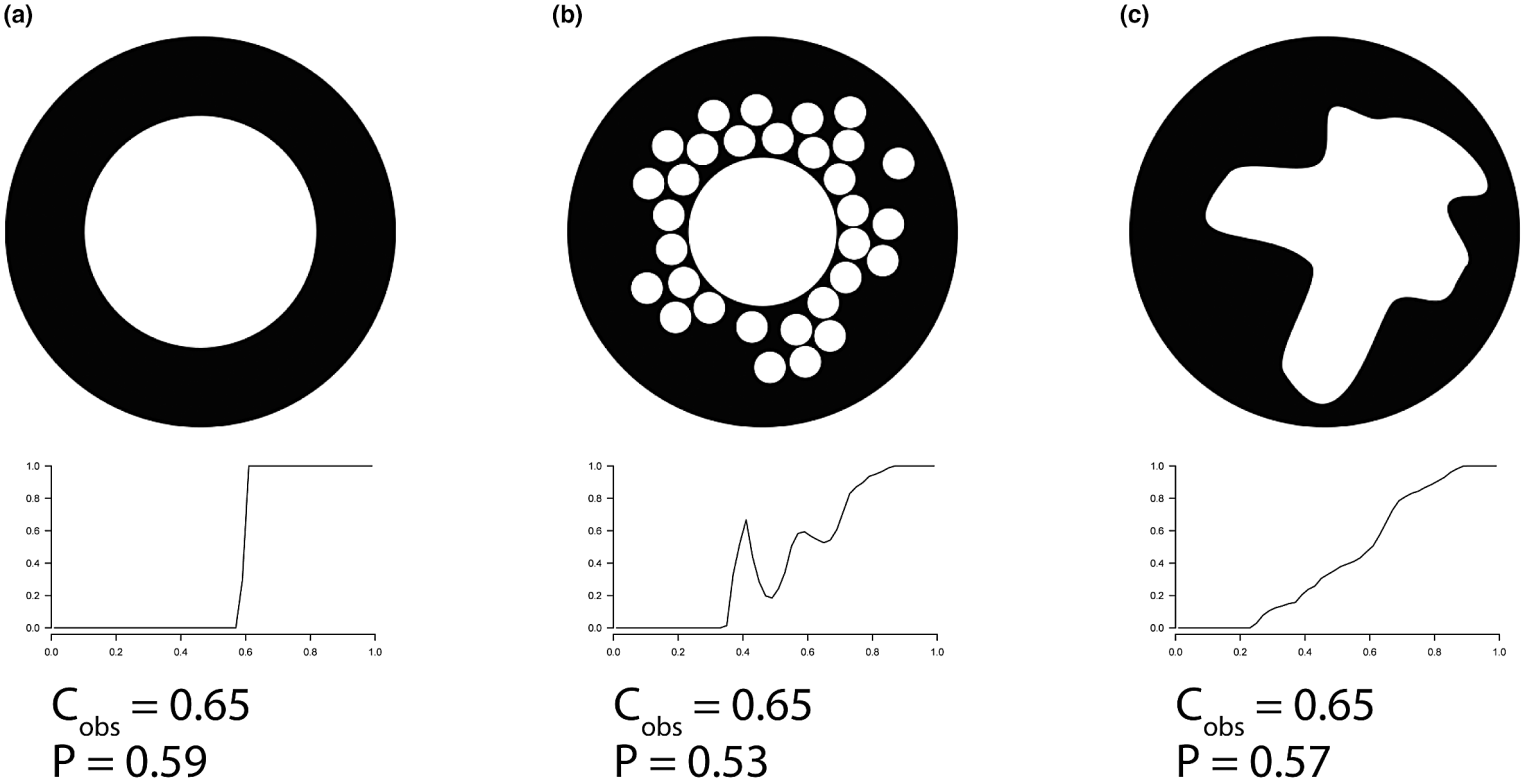
Schematic representation of bone cross‐sections with fixed bone compactness (C_obs_) and variable P (distance from the centre of the section of the medullocortical transition), and their associated compactness profile as obtained with BoneProfileR (Gônet et al., 2022).

A possible hypothesis to explain this decorrelation between the parameter P and bone compactness is based on the way the cortex is structured (Figure 8). Indeed, one way for P to vary without altering compactness implies the presence of more or less cancellous bone (Figure 8b). For example, if P decreases, C_obs_ mechanically increases, unless the cortical bone becomes spongier, in which case C_obs_ remains constant. The taxa in our sample do not have particularly spongy bones, with the exception of turtles, but there are only two turtles. Another plausible hypothesis is that the cortex shows variations in thickness (Figure 8c). As a reminder, compactness is calculated from the centre of the section in increasingly larger concentric circles. If the thickness of the cortex is not the same all around the section, with thinner and thicker areas, this considerably extends the overall transition zone between the medulla and the cortex (S), and the global P could vary while compactness remains constant. The RSSD and RPSD parameters (the standard deviation associated with RS and RP, respectively) allow us to study the angular variation of S and P. However, the results of the phylogenetic ANOVA are not significant (Table 4). This should be studied more carefully.

### 4.2 Functional ecology and body mass effects

The impact of the presence of varanids and iguanids on the scores of our models could be related to their body mass. Indeed, varanids and iguanids are the heaviest among our sample of lepidosaurians. Furthermore, our analyses show that at least one axis of our models is significantly impacted by the body mass of the individuals. Body mass increases for all functional ecology groups, as individuals have larger values on the second axis of model 1 (Figure 7b). Body mass also increases, more dramatically, as individuals have higher values on the first axis of model 2 (Figure 7c).

Body mass also appears to impact the first axis of model 1, except that there is an interaction between the regression slopes. Thus, the effect of body mass is not the same for all functional ecology groups (Figure 7a). Indeed, body mass increases for arboreal and terrestrial species as individuals have lower values on this axis. This also appears to be the case for fossorial species, even though the sample size is limited for this ecological group (only 3 individuals). However, the opposite relationship is observed for aquatic species: their body mass decreases as individuals present lower values. This seems to be the result of mixing semi‐aquatic birds and crocodylians in the same functional ecology group. Considered separately, birds and crocodylians seem to follow the same trend as the other groups. The same conclusion can be drawn for the second axis of model 2 (Figure 7d), despite the fact that the overall effect of body mass is only close to significance: mass increases as individuals have lower values on this axis, except for aquatic species. But if we separate birds and crocodylians, we recover the above relationship.

Thus, the body mass of individuals does seem to have an effect on our models. This is not surprising since these models are based on parameters such as the cross‐sectional perimeter which is significantly related to body mass (Table 6). Locomotor mode, posture and body mass are generally related (Biewener, 2005; Houssaye, Waskow, et al., 2016; Pintore et al., 2022). Nevertheless, our models show a high discriminating power based only on easily measurable parameters, which is particularly important in the case of fossils, and yield decent predictions for extinct taxa, which was one of the goals of our study. In general, while body mass is strongly associated with locomotion and posture, it is not sufficient. For example, with our locomotion model, many species in the sample have equivalent body mass, but not the same mode of locomotion, for example *Gypaetus barbatus* (biped; 5.606 kg) and *Chelydra serpentina* (quadruped; 5.17 kg) in the sample. Similarly, while we are unable to say that the apparent discrimination between crouched and erect birds along the first axis of the postural model (Figure 6b) is not entirely due to body mass, our model seems much more reliable when considering the discrimination along the second axis between parasagittally locomoting taxa on the one hand and sub‐parasagittally locomoting and sprawling taxa on the other. Furthermore, we showed that when only the parameters associated with body mass are retained in the models, the classification performance decreases drastically (Table 8). Therefore, we conclude that femoral geometric and microanatomical parameters contain a functional signal that a multivariate quantitative approach such as PFDA can effectively exploit.

The mean values of the sampled individuals on the different axes of our models are never significantly different between functional ecologies. The *p*‐value is close to significance for the second axis of our first model; it is significant at the 0.05 threshold with tree 22. Unfortunately, our post hoc tests are all non‐significant and thus prevent us from determining which group is different. This could be due to several reasons: (1) a lack of statistical power caused by overly small sample size; (2) functional ecology is coded as a four‐level factor, which implies more comparisons that increase the penalty of each *p*‐value that corrections could not overcome; (3) the overall signal is not strong enough; (4) all of these reasons at once. In any case, we can assume that the effect of functional ecology on our models, if any, is very small.

### 4.3 Palaeobiological implications

The captorhinid *Labidosaurus* is inferred as quadrupedal with our first model, which was expected given that *Labidosaurus*, and Captorhinidae in general, are usually presented as quadrupedal sprawlers on the basis of osteological, muscular and ichnological evidence (Heaton & Reisz, 1980; Holmes, 2003; Logghe et al., 2021; Sumida, 1989). However, it is inferred as erect with our second model, which is more surprising, and probably wrong. When we remove the eccentricity of the section (Ecc) from the model parameters, it comes out 'semi‐erect', which might be considered plausible. Indeed, similarities may exist between Captorhinidae and Crocodylia, at least from an ichnological perspective, with digit and tail drag tracks suggesting a similar step cycle (Logghe et al., 2021), although the mechanics of the limb are most likely different.

The locomotion in *Euparkeria* has been controversial for decades. *Euparkeria* has been interpreted as a facultative biped on the basis of its limb proportions (Ewer, 1965), although this has been questioned. Remes (2008) suggested that it could have adopted a sprawling or 'semi‐erect' posture. Inferences with our first model produced *Euparkeria* as a quadruped. Our model included only a small number of facultative bipeds but still has a good overall classification score, so we are fairly confident in this result, which is in agreement with other recent work (Demuth et al., 2020; Pintore et al., 2022). With our second model, *Euparkeria* is classified as either erect or with a sprawling posture, two apparently contradictory but plausible hypotheses. Indeed, the hind limb of *Euparkeria* displays a mosaic of ancestral and derived characters. In particular, the pelvis suggests a slightly more erect posture, while the ankle evokes a more sprawling posture (Demuth et al., 2020).

Among dinosaurians, the case of *Marasuchus* is reminiscent of that of *Euparkeria*. It has long been accepted as a biped based on a wide variety of evidence such as limb proportions (Benton, 2006, p. 20) as well as centre of mass and body shape (Bishop et al., 2020) and 3D femur dimensions (Pintore et al., 2022). While it resembles later dinosaurs in several respects, notably regarding the pelvis and the proximal part of the femur, the ankle retains some ancestral features (Sereno & Arcucci, 1994). *Marasuchus* is inferred as a facultative biped with our first model. This implies that it spent most of its time on four legs, which is incompatible with its limb proportions and other aspects of its morphology, unless it adopted a sprawling posture, as our second model suggests. Indeed, the facultative bipeds in our sample have the shortest forelimbs relative to the hind limbs, as well as a long, heavy tail for balancing during bipedal running (Snyder, 1949). *Marasuchus* also has a very long tail (Sereno & Arcucci, 1994). If correct, our inferences would represent a significant change in the way we envision locomotion in this animal (cf. Cuff et al., 2022). However, this is most likely due to a lower discriminatory power of our model. Still, a better characterisation of the traits associated with facultative bipedalism in early archosaurs is warranted (Grinham et al., 2019).

The first dinosaurs were bipedal with an erect posture. Dinosauria is a very interesting clade since it is the only known reptilian clade to have experienced several reversions to a quadrupedal state (Barrett & Maidment, 2017). *Plateosaurus* is among the earliest described dinosaurs and has been assigned many different postures over time, from erect bipedalism to facultative quadrupedalism to strict sprawling quadrupedalism (Christian et al., 1996; Jaekel, 1910; von Huene, 1926). Recently, numerous studies have concluded that it was strictly bipedal (e.g. Bishop et al., 2020; Bonnan & Senter, 2007; Mallison, 2010; Pintore et al., 2022). Our results corroborate this modern consensus, since *Plateosaurus* is inferred as an erect biped in all cases.

*Dysalotosaurus* is inferred to be bipedal and erect. The indeterminate hypsilophodontid is inferred bipedal with our first model. It is inferred as a sprawler with the parameter configuration including eccentricity with the second model. This result is obviously due to a taphonomic artefact. It is inferred as erect when eccentricity is excluded from the model. These results show that these animals do not exhibit, at least when considering femur geometry and microanatomy, the characters associated with quadrupedalism in the geologically more recent ornithopod taxa.

Based on limb proportions, it has been suggested that early crocodylomorphs such as *Terrestrisuchus* were bipedal (Seymour et al., 2004), but a recent study employing 3D geometric morphometrics of the femur suggested that it instead was quadrupedal (Pintore et al., 2022), in agreement with prior morphological analyses (e.g. Russell & Wu, 1997), including quantitative analysis of limb proportions (Kubo & Kubo, 2012). Our model infers it to be bipedal or facultatively bipedal. When varanids and iguanids are excluded from the model, *Terrestrisuchus* is inferred as strictly bipedal. Bipedalism evolved independently in dinosaurs and Pseudosuchia such as *Poposaurus* (Gauthier et al., 2011; Kubo & Kubo, 2012). Thus, a bipedal posture in *Terrestrisuchus* remains open to interpretation. Furthermore, because of its femoral morphology, it is thought to have adopted a strictly erect posture (Crush, 1984), which is purely inconsistent with the inferences of our second model (sprawler). This may be due to taphonomic artefact or low discriminative power of our model. In any case, while Triassic crocodylomorphs are broadly accepted as having had more erect postures (Crush, 1984; Russell & Wu, 1997; Sereno, 1991), they deserve further morphofunctional work to better understand whether bipedalism evolved in this group or not (see also Cuff et al., 2022).

### 4.4 Limitations of the method

*Composition of the sample*. Our current sample is composed mainly of birds (61%), which is a concern. However, if we consider the taxonomic representation of each extant reptilian clade—there are nearly 10,000 extant species of birds (Jetz et al., 2012), about 5,000 species of limbed squamates (Brandley et al., 2008), about 350 turtle species (Thomson et al., 2021), and around 25 species of crocodylians (Brochu, 2003)—birds are only moderately over‐represented. Also, a problematic over‐representation of birds would have resulted in a very good classification score for the overall models, but this would be an artefact caused by the good score of the birds themselves (since the majority of observations are birds, we would expect them to weigh much more heavily on the overall model score), while non‐avian taxa would be poorly discriminated, attracted by the bird ‘block’. Here, the non‐avian taxa, despite the small sample size, show decent rates of correct classification, for example quadrupeds (locomotion model: 71%) and sprawling species (postural model: 97%). Nevertheless, there is no doubt that a larger sample of lepidosaurs, turtles and crocodylians would allow us to refine the predictions of our model.

*Taphonomic impact*. When the parameter Ecc is excluded from our postural model, inferences for extinct taxa appear to improve. This is relevant because cross‐sectional eccentricity is strongly influenced by taphonomic processes. A crushed or even plastically deformed diaphysis could largely impact the eccentricity of the cross‐section. In *Euparkeria*, the angle between the proximal and distal epiphyses is unusually high (Pintore et al., 2022). This may result from taphonomic processes, which may have flattened the diaphysis. The *Labidosaurus* femur, however, does not appear to have been crushed or deformed. In the end, even after excluding these parameters from our model, some inferences remain surprising. This could be due to the model itself. Regardless, one should be cautious when considering parameters that might be influenced by taphonomic processes.

**FIGURE 9:**
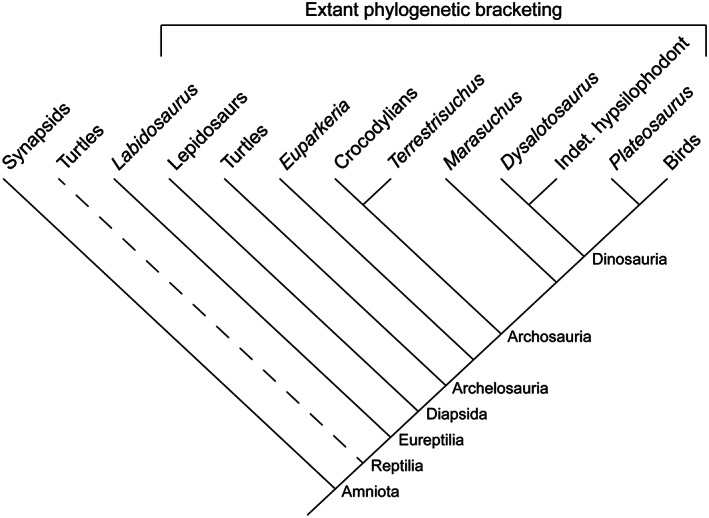
Extant phylogenetic bracketing (Witmer, 1995) applied to the taxa in this study. Depending on the position of turtles in the tree, *Labidosaurus* is contained within the bracket or not. Dotted branch, turtles in parareptiles as sister taxa to Eureptilia, as proposed by Laurin & Reisz (1995).

*Palaeobiological inferences outside the extant phylogenetic bracket. Euparkeria*, *Terrestrisuchus* and *Marasuchus* are contained within the extant phylogenetic bracket in the sense of Witmer (1995), that is they are ‘framed’ by two clades with extant representatives (lepidosaurs and archosaurs) and which are their closest sister taxa (Figure 9). Therefore, our current data set is relevant for making palaeobiological inferences about these taxa. The small sample size of lepidosaurs does not mean that no inferences can be made. The same applies to the dinosaurs *Plateosaurus*, *Dysalotosaurus* and the indeterminate hypsilophodont. The only taxon outside the bracket—if turtles are indeed among Diapsida as suggested by most molecular phylogenies (see Figure 9)—is *Labidosaurus*. For it to remain in the bracket, we would have to include mammals in our sample, which we believe would be irrelevant since synapsids and reptiles show very different locomotor patterns. As is too often the case in palaeontology, we lack a good comparison in nature today. The diversity of extant reptiles remains the best source of comparison to try to understand the biology of early reptiles such as *Labidosaurus*.

## 5 CONCLUSIONS

We showed from mid‐diaphyseal femoral thin sections that there was a significant difference in the P parameter (distance from the centre of the section of the medullocortical transition) between quadrupeds and bipeds—implying a larger medullary cavity in the latter—but not in bone compactness (C_obs_). A possible reason for this result could be due to variations in cortical thickness.

The existence of a phylogenetic signal in the geometric and microanatomical parameters justifies the use of phylogenetic flexible discriminant analyses (PFDA). In general, our models show good statistical power to discriminate postures and modes of locomotion with our sample of extant species. The exclusion of varanids and iguanids from our sample increases the score of our models. Although body mass is significantly related to some parameters in the model and to the model axes (but not functional ecology), the models seem to have had some success in inferring the posture and locomotion in extinct animals. The fact that few parameters were used in our models is good for palaeontologists because most of the time, they work with incomplete material. However, it seems necessary to consider the impact of taphonomy on the fossils because accurate assessment of some parameters such as the eccentricity of the cross‐section requires undeformed bones. The fact that we used 100 phylogenetic trees allows us to take into account the phylogenetic uncertainty. While the literature mentions that even a small variation in lambda can have an impact on the results, the impact is very small here.

Our models produced some plausible inferences. The captorhinid *Labidosaurus hamatus* is inferred to have been a 'semi‐erect' quadruped, while the dinosaurs in our sample are inferred to have been erect bipeds, as expected. *Euparkeria capensis* is inferred to be a quadrupedal sprawler, but this could be seen in the light of the fact that the hind limb of *Euparkeria* shows a mosaic of ancestral and derived characters. *Marasuchus lilloensis* is inferred to be a facultative biped with a sprawling posture, but we admit that this is highly contradictory of other analyses from multiple lines of evidence and may be an artefact of our analysis. Finally, *Terrestrisuchus* is inferred to have been bipedal. There has been less functional analysis (especially quantitative) of the limbs of early Crocodylomorpha as compared with *Marasuchus*, so the question of locomotor mode in these taxa remains ambiguous.

Our study sheds light on the evolution of the enigmatic locomotion of various early reptiles. Our models and methods could be used by palaeontologists to infer the posture and locomotion of other extinct reptiles, especially when combined with other lines of evidence. In the future, increasing our sample size with more sprawling taxa; for example, turtles and lepidosaurs; and more facultatively bipedal species as well as fossils with ‘known’ locomotion and posture, could help improve the discriminatory performance of our model.

## AUTHOR CONTRIBUTIONS

J.G. collected the data, designed the study, performed the analyses, interpreted the results and wrote the manuscript. J.B. designed the analyses. M.G. designed the analyses. J.R.H. collected the data and interpreted the results. M.L. collected the data, designed the study and interpreted the results. All authors reviewed and approved the final manuscript.

## Supporting information


Supporting Information S1

## Data Availability

The data that support the findings of this study are included in this published article and its Supporting Information files.
