# Supplementary material for: Locomotor and postural diversity among reptiles viewed through the prism of femoral microanatomy: Palaeobiological implications for some Permian and Mesozoic taxa
Source: J Anat. 2023 Feb 17;242(5):891–916. doi: 10.1111/joa.13833 (PMC10093171; doi:10.1111/joa.13833)
Supplement: Supplementary file 1 — Supporting Information S1 [file JOA-242-891-s001.zip › Appendix.docx]

GÔNET ET AL. Locomotor and postural diversity among reptiles: palaeobiological implications

SUPPLEMENTARY INFORMATION

1. Phylogenetic flexible discriminant analyses: alternative models

Varanids and iguanids impacted our locomotion model. They were excluded from the dataset. The percentage of correct classification (PCC) with the training dataset and with the test dataset (cross-validation) is 94% and 91% respectively. The PCCs corresponded to an average value calculated from 100 phylogenetic trees.

*Plateosaurus engelhardti*, *Terrestrisuchus gracilis*, the indeterminate hypsilophodontid and *Dysalotosaurus lettowvorbecki* are inferred as bipedal; *Marasuchus lilloensis* is inferred as facultatively bipedal; *Euparkeria capensis* and *Labidosaurus hamatus* are inferred as quadrupedal. Figure 1 shows the corresponding phylogenetic discriminant space produced from the axes derived from PFDA with tree 1.


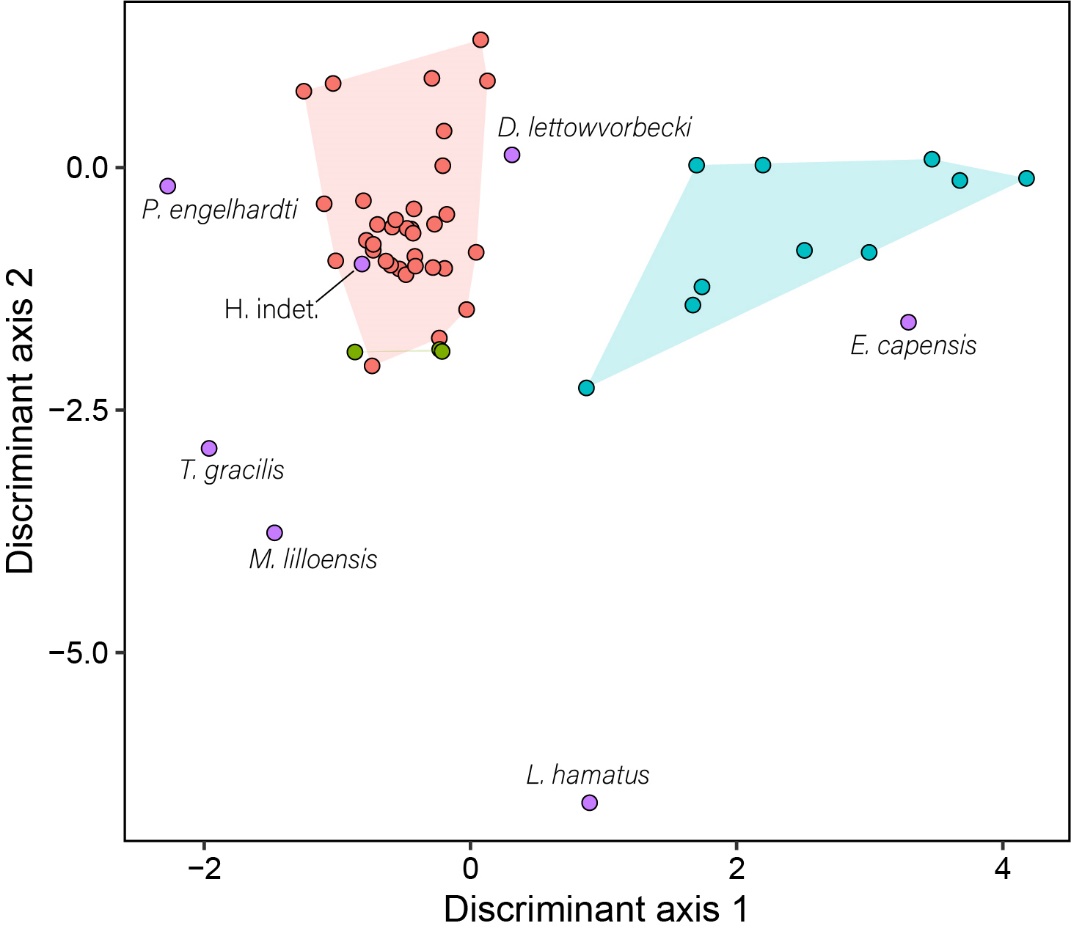


**FIGURE 1** Morphometric separation of reptiles according to their locomotion, as shown by phylogenetic discriminant spaces generated by PFDA on femoral geometric and microanatomical data with tree 1 (varanids and iguanids excluded). The red and blue circles represent bipedal and quadrupedal species, respectively. The green circles represent facultative bipedal species. The purple circles represent the species for which we want to infer the mode of locomotion. Inferences: biped, Plateosaurus engelhardti, Terrestrisuchus gracilis, the indeterminate hypsilophodontid, Dysalotosaurus lettowvorbecki; facultative biped, Marasuchus lilloensis; quadruped, Euparkeria capensis, Labidosaurus hamatus.

Varanids and iguanids also impacted our postural model. They were excluded from the dataset. The percentage of correct classification (PCC) with the training dataset and with the test dataset (cross-validation) was 91% and 81% respectively. The PCCs corresponded to an average value calculated from 100 phylogenetic trees.

*Labidosaurus hamatus*, *Euparkeria capensis*, *Plateosaurus engelhardti* and *Dysalotosaurus lettowvorbecki* were inferred as erect; *Marasuchus lilloensis* and *Terrestrisuchus gracilis* were inferred as sprawlers; the indeterminate hypsilophodontid was inferred as parasagittal crouched. Figure 2 shows the corresponding phylogenetic discriminant space produced from the axes derived from PFDA with tree 1.


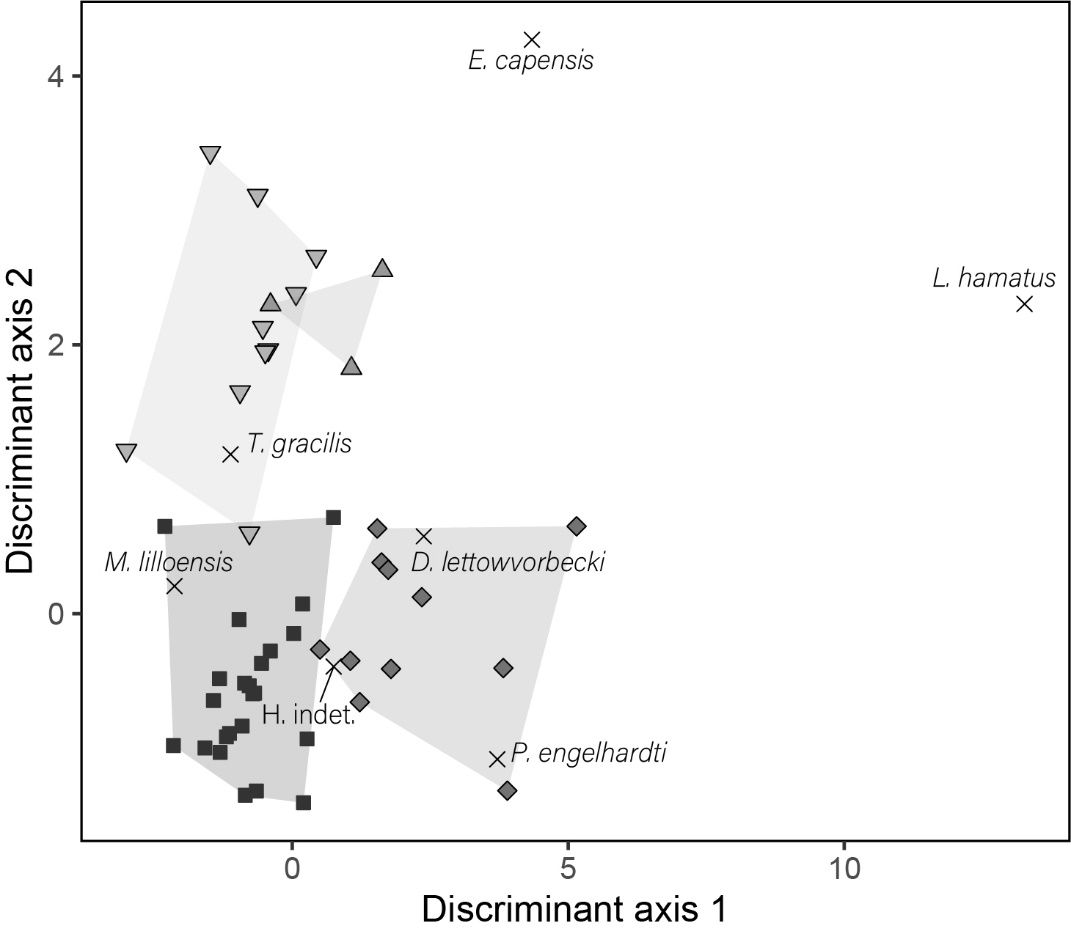


**FIGURE 2** Morphometric separation of reptiles according to their posture, as shown by phylogenetic discriminant spaces generated by PFDA on femoral geometric and microanatomical data with tree 1 (varanids and iguanids excluded). The squares represent parasagittal crouched species. The inverted triangles and the triangles represent sprawling and semi-erect species, respectively. The diamonds represent erect species and the crosses the species for which we inferred the posture. Inferences: sprawling, Marasuchus lilloensis, Terrestrisuchus gracilis; parasagittal crouched, the indeterminate hypsilophodontid; erect, Dysalotosaurus lettowvorbecki, Plateosaurus engelhardti, Euparkeria capensis, Labidosaurus hamatus.

We excluded the eccentricity of the cross-section from the parameters of our postural model in order to take into account a possible taphonomic bias. The parameters used were SR, C_obs_, RS and Pe_min_. They corresponded to the best combination of parameters identified through cross-validation procedures. The percentage of correct classification (PCC) with the training dataset and with the test dataset (cross-validation) was 73% and 60% respectively; 81% and 70% without varanids and iguanids in the dataset. The PCCs corresponded to an average value calculated from 100 phylogenetic trees.

*Euparkeria capensis*, *Terrestrisuchus gracilis* and *Marasuchus lilloensis* were inferred as sprawlers; *Labidosaurus hamatus* was inferred as semi-erect. *Plateosaurus engelhardti*, *Dysalotosaurus lettowvorbecki* and the indeterminate hypsilophodontid were inferred as erect. Inferences were the same with or without varanids and iguanids in the training dataset. Figure 3 shows the corresponding phylogenetic discriminant space produced from the axes derived from PFDA with tree 1.


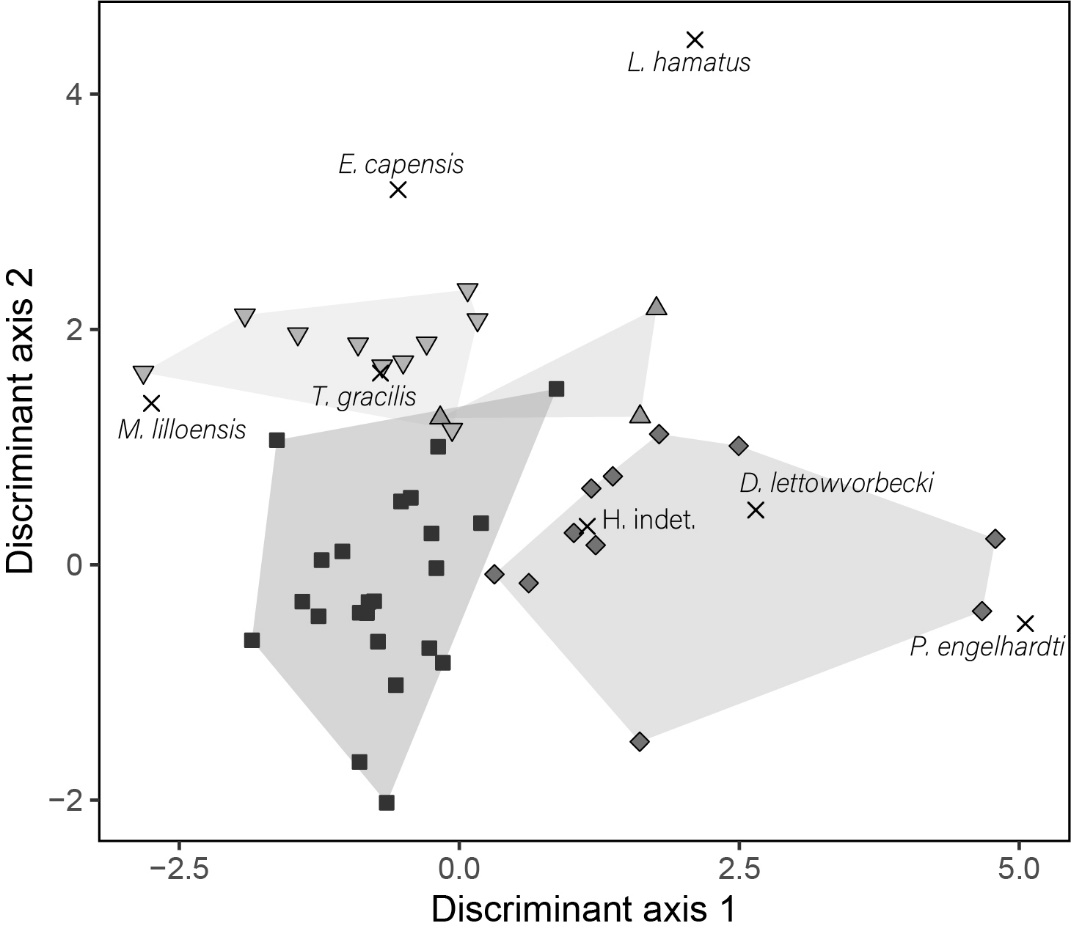


**FIGURE 3** Morphometric separation of reptiles according to their posture, as shown by phylogenetic discriminant spaces generated by PFDA on femoral geometric and microanatomical data with tree 1 (eccentricity excluded; varanids and iguanids excluded). The squares represent parasagittal crouched species. The inverted triangles and the triangles represent sprawling and semi-erect species, respectively. The diamonds represent erect species and the crosses the species for which we inferred the posture. Inferences: sprawling, Euparkeria capensis, Marasuchus lilloensis, Terrestrisuchus gracilis; semi-erect, Labidosaurus hamatus; erect, Plateosaurus engelhardti, Dysalotosaurus lettowvorbecki, the indeterminate hypsilophodontid.

1. Phylogenetic ANOVA: post-hoc tests

The P parameter was the only one that was significantly different between modes of locomotion and postures. Table 1 summaries the post-hoc test results.

**TABLE 1** List of adjusted p-values for each pairwise comparison of locomotor modes and postures for the parameter P. P-values are averaged over 100 phylogenetic trees.

| **Model** | **Pairwise comparison** | **Mean adjusted p-value** |
| --- | --- | --- |
| Locomotion | $Biped vs Facultative biped$ | 0.958 |
|  | $Biped vs Quadruped$ | 0.028* |
|  | $Facultative biped vs Quadruped$ | 0.16 |
| Posture | $Crouched vs Erect$ | 0.313 |
|  | $Crouched vs Semi-erect$ | 0.022* |
|  | $Crouched vs Sprawling$ | 0.754 |
|  | $Erect vs Semi-erect$ | 0.056 |
|  | $Erect vs Sprawling$ | 1 |
|  | $Semi-erect vs Sprawling$ | 0.632 |

1. Post-hoc tests for tree 22

Functional ecology was significantly associated with the second axis of the postural model with tree 22 only. A summary of the post-test results is presented in Table 2.

**TABLE 2** List of adjusted p-values for each pairwise comparison of functional ecologies with tree 22.

| **Pairwise comparison** | **Adjusted p-value** |
| --- | --- |
| $Aq vs Ar$ | 0.164 |
| $Aq vs Fo$ | 0.279 |
| $Aq vs Te$ | 0.728 |
| $Ar vs Fo$ | 0.728 |
| $Ar vs Te$ | 0.089 |
| $Fo vs Te$ | 0.180 |

Abbreviations: Aq, semi-aquatic; Ar, arboreal; Fo, fossorial; Te, terrestrial.
